# Emerging nitric oxide gas‐assisted cancer photothermal treatment

**DOI:** 10.1002/EXP.20230163

**Published:** 2024-03-24

**Authors:** Shuang Liang, Yufei Liu, Hongquan Zhu, Guangfu Liao, Wenzhen Zhu, Li Zhang

**Affiliations:** ^1^ Department of Radiology, Tongji Hospital, Tongji Medical College Huazhong University of Science and Technology Wuhan China; ^2^ College of Material Engineering Fujian Agriculture and Forestry University Fuzhou China; ^3^ Department of Critical Care Medicine Shenzhen Hospital Southern Medical University Shenzhen Guangdong China; ^4^ Department of Chemistry City University of Hong Kong Kowloon Hong Kong SAR China

**Keywords:** cancer nanotheranostics, gas therapy, nitric oxide, photothermal therapy

## Abstract

Photothermal therapy (PTT) has garnered significant attention in recent years, but the standalone application of PTT still faces limitations that hinder its ability to achieve optimal therapeutic outcomes. Nitric oxide (NO), being one of the most extensively studied gaseous molecules, presents itself as a promising complementary candidate for PTT. In response, various nanosystems have been developed to enable the simultaneous utilization of PTT and NO‐mediated gas therapy (GT), with the integration of photothermal agents (PTAs) and thermally‐sensitive NO donors being the prevailing approach. This combination seeks to leverage the synergistic effects of PTT and GT while mitigating the potential risks associated with gas toxicity through the use of a single laser irradiation. Furthermore, additional internal or external stimuli have been employed to trigger NO release when combined with different types of PTAs, thereby further enhancing therapeutic efficacy. This comprehensive review aims to summarize recent advancements in NO gas‐assisted cancer photothermal treatment. It commences by providing an overview of various types of NO donors and precursors, including those sensitive to photothermal, light, ultrasound, reactive oxygen species, and glutathione. These NO donors and precursors are discussed in the context of dual‐modal PTT/GT. Subsequently, the incorporation of other treatment modalities such as chemotherapy (CHT), photodynamic therapy (PDT), alkyl radical therapy, radiation therapy, and immunotherapy (IT) in the creation of triple‐modal therapeutic nanoplatforms is presented. The review further explores tetra‐modal therapies, such as PTT/GT/CHT/PDT, PTT/GT/CHT/chemodynamic therapy (CDT), PTT/GT/PDT/IT, PTT/GT/starvation therapy (ST)/IT, PTT/GT/Ca^2+^ overload/IT, PTT/GT/ferroptosis (FT)/IT, and PTT/GT/CDT/IT. Finally, potential challenges and future perspectives concerning these novel paradigms are discussed. This comprehensive review is anticipated to serve as a valuable resource for future studies focused on the development of innovative photothermal/NO‐based cancer nanotheranostics.

## Graphical abstract

A comprehensive review summarizes recent advancements in nitric oxide (NO) gas‐assisted cancer photothermal treatment. It commences by providing various types of NO donors/precursors for dual‐modal therapy. Subsequently, chemotherapy, photodynamic therapy, alkyl radical therapy, radiation therapy, immunotherapy, chemodynamic therapy, starvation therapy, Ca^2+^ overload, and ferroptosis are incorporated for triple‐ and tetra‐modal treatment.

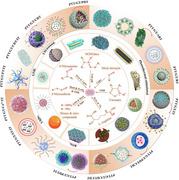

## 1 INTRODUCTION

Cancer, characterized by its aggressive nature and substantial morbidity and mortality rates, poses a formidable challenge to human health.^[^ ^1^, ^2^, ^3^ ^]^ In clinical practice, conventional treatment modalities such as surgery, chemotherapy (CHT), and radiotherapy (RT) have been widely utilized to combat diverse forms of cancer.^[^ ^4^, ^5^, ^6^ ^]^ Nevertheless, these conventional therapies are burdened with inherent limitations, including invasiveness, severe adverse effects, and the development of drug resistance, ultimately resulting in unfavorable and potentially fatal outcomes.^[^ ^7^, ^8^ ^]^

Photothermal therapy (PTT), activated by near‐infrared (NIR) light within the 700–1350 nm range, utilizes diverse photothermal agents (PTAs) to induce localized heat at targeted sites, thereby ablating tumors.^[^ ^9^, ^10^, ^11^ ^]^ PTT has garnered significant attention in preclinical research over the past decade due to its minimally invasive nature, high tumor specificity, favorable therapeutic outcomes, and reduced side effects.^[^ ^12^, ^13^ ^]^ Generally, PTAs can be categorized into inorganic nanomaterials (e.g., noble metals, metal sulfides, and two‐dimensional Mxenes) and organic nanomaterials (e.g., small organic molecules and conjugated polymers), and the effectiveness of PTT heavily relies on the photothermal conversion efficiency (PCE) exhibited by these PTAs.^[^ ^14^, ^15^, ^16^, ^17^, ^18^, ^19^, ^20^ ^]^ To further enhance PTT efficiency, recent efforts have focused on developing near‐infrared‐II (NIR‐II) PTAs. In comparison to NIR‐I (700–1000 nm), NIR‐II light (1000–1350 nm) offers several advantages, including improved tissue‐penetrating capacity, reduced light‐tissue interactions, and higher maximum permissible exposure (MPE) values (1, 0.72, and 0.33 W cm^−2^ for 1064, 980, and 808 nm laser, respectively).^[^ ^21^, ^22^, ^23^ ^]^

Despite significant advancements, the efficacy of single‐modal PTT in achieving complete tumor eradication still remains limited due to various constraints. One such limitation arises from the existence of inherent or acquired resistance to the generated heat exhibited by certain tissues or cells. This heat resistance can be attributed to various factors, including intrinsic thermotolerance, elevated expression of heat shock proteins (HSPs), altered heat dissipation mechanisms, or enhanced repair mechanisms, resulting in reduced susceptibility to thermal damage and limiting the effectiveness of PTT. Moreover, localized heat during PTT can induce cellular stress, triggering the activation of various cellular responses. Autophagy activation, as a protective mechanism, eliminates damaged proteins, organelles, or other cellular components affected by thermal stress.^[^ ^24^, ^25^ ^]^ Consequently, it becomes imperative to explore cooperative paradigms that integrate PTT with other modalities to enhance the anticancer efficacy.^[^ ^26^, ^27^ ^]^

Gas therapy (GT) has emerged as a novel treatment modality employing diverse gasotransmitters to induce tumor cell death.^[^ ^28^, ^29^ ^]^ In contrast to conventional CHT or RT, GT provides a greener therapeutic approach with minimal adverse toxicity.^[^ ^30^, ^31^ ^]^ Among the three main gasotransmitters (i.e., nitric oxide (NO), carbon monoxide (CO), and hydrogen sulfide (H_2_S)) investigated for GT, NO has received significant attention due to its multifaceted roles in cancer biology and potential as a therapeutic agent.^[^ ^32^, ^33^ ^]^ Although naturally produced NO exhibits greater potential for cancer therapy than exogenously synthesized drugs, its therapeutic effects are highly concentration‐dependent.^[^ ^34^, ^35^ ^]^ Recent studies have demonstrated that low concentrations of NO can promote tumor growth, while higher concentrations (>1 µm) can induce tumor cell apoptosis through oxidation or nitrosation of mitochondria and DNA.^[^ ^36^ ^]^ Given its short half‐life (≈5 s) and limited diffusion radius (40−200 µm), the development of multifunctional nanoplatforms capable of targeted delivery and controlled release of an optimal amount of NO has become a research focus.^[^ ^37^, ^38^, ^39^ ^]^ Over the past decade, various endogenous (e.g., pH, glutathione (GSH), and hydrogen peroxide (H_2_O_2_)) and exogenous (e.g., light, X‐ray, and ultrasound) stimuli have been extensively investigated to trigger NO release from different NO donors and precursor.^[^ ^40^, ^41^, ^42^, ^43^, ^44^, ^45^, ^46^, ^47^, ^48^, ^49^ ^]^ Among these stimuli, NIR light offers superior advantages as it allows for on/off NO generation and on‐demand NO release at specific tumor sites, which can be precisely controlled by laser irradiation and output energy.^[^ ^50^, ^51^ ^]^

The combination of PTT and NO‐mediated GT presents a multifaceted approach to cancer treatment that offers numerous advantages. First, NO gas has the ability to induce vasodilation, which can enhance blood flow and oxygenation in the tumor, enabling better delivery of PTAs to deeper regions of the tumor. This improved penetration and distribution led to more effective tumor destruction throughout the entire tumor volume. Second, PTT is localized to the tumor site, minimizing damage to healthy tissues. NO gas can also be targeted specifically to the tumor microenvironment (TME), reducing off‐target effects on healthy cells and tissues. This targeted approach improves the safety profile of the combined treatment. Third, NO gas can sensitize tumor cells to PTT by inhibiting protective autophagy, promoting more efficient tumor cell death via combined treatment. Fourth, NO gas has the potential to modulate HSPs and reduce heat resistance. By targeting HSPs, NO can enhance the susceptibility of tumors to PTT‐induced hyperthermia, allowing for improved tumor destruction and overcoming resistance. Fifth, PTT is capable of inducing immunogenic cell death (ICD), leading to the release of tumor antigens and the activation of immune cells. NO gas, with its immunomodulatory properties, can further stimulate the immune system and promote an anti‐tumor immune response. This combined effect is able to improve the body's ability to recognize and eliminate cancer cells beyond the direct tumor destruction achieved by PTT. Sixth, the integration of PTT and NO gas can synergize with other treatment modalities, such as CHT, making cancer cells more susceptible to the cytotoxic effects of chemotherapeutic drugs.^[^ ^39^, ^52^, ^53^, ^54^, ^55^, ^56^, ^57^ ^]^

**SCHEME 1:**
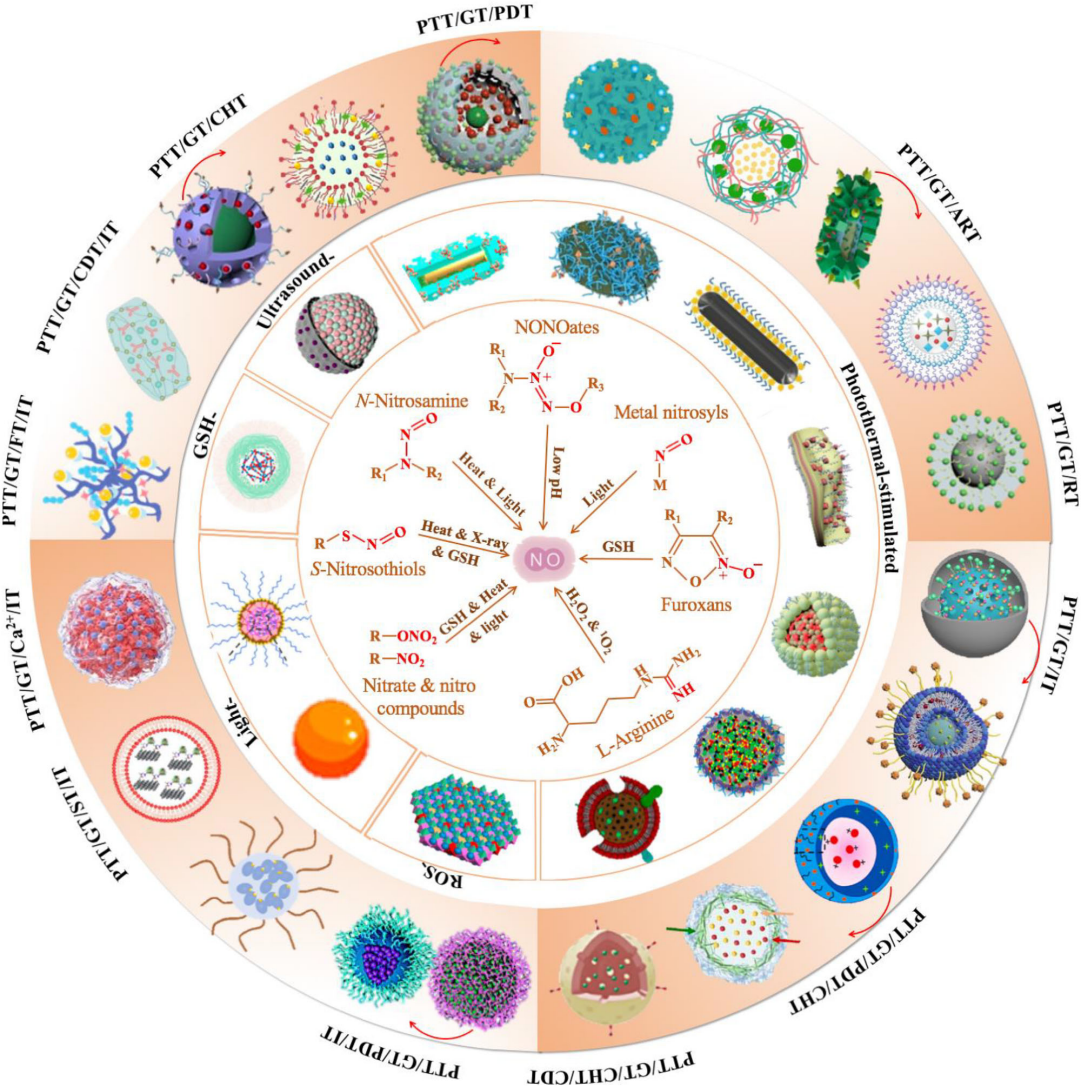
Schematic illustration of various photothermal/NO‐generating nanoplatforms and different NO donors/precursors with corresponding NO‐releasing mechanisms.

Up to now, several reviews have focused on describing diverse NO‐releasing nanosystems and NO‐based cancer theranostics.^[^ ^31^, ^32^, ^41^, ^44^, ^48^, ^51^, ^58^, ^59^, ^60^, ^61^, ^62^, ^63^, ^64^, ^65^, ^66^, ^67^, ^68^, ^69^, ^70^, ^71^, ^72^, ^73^, ^74^, ^75^, ^76^, ^77^ ^]^ In this work, we provide a comprehensive summary of emerging NO‐assisted photothermal nanomedicines, the significant advances of which have not been reported yet to the best of our knowledge (Scheme 1). It begins by discussing various types of NO donors and precursors, including photothermal‐, light‐, ultrasound‐, reactive oxygen species (ROS)‐, and GSH‐sensitive ones, within the context of dual‐modal PTT/GT. Subsequently, the incorporation of other treatment modalities such as CHT, photodynamic therapy (PDT), alkyl radical therapy (ART), radiation therapy (RT), and immunotherapy (IT) for triple‐modal therapy is introduced. Furthermore, tetra‐modal therapies such as PTT/GT/CHT/PDT, PTT/GT/CHT/chemodynamic therapy (CDT), PTT/GT/PDT/IT, PTT/GT/starvation therapy (ST)/IT, PTT/GT/Ca^2+^ overload/IT, PTT/GT/ferroptosis (FT)/IT, and PTT/GT/CDT/IT are also discussed. Finally, the potential challenges and future perspectives of these novel paradigms are addressed.

## 2 DUAL‐MODAL PTT/GT

Conventional PTT is able to activate protective autophagy that limits the antitumor efficiency, while heat resistance of tumors with upregulated HSPs is also a critical issue for PTT.^[^ ^78^, ^79^, ^80^, ^81^, ^82^ ^]^ Apart from being involved in angiogenesis, apoptosis, and immuno responses, NO gas has recently proven to inhibit the protective autophagy and HSP expression of cancer cells, resulting in enhanced photothermal efficacy. Therefore, the integration of PTT with NO gas‐mediated GT is of great significance. In this section, we present typical NO donors and precursors, such as those triggered by photothermal effects, light, ultrasound, ROS, and GSH, to enhance PTT efficacy.

### 2.1 Photothermal‐sensitive NO donors for dual‐modal therapy

When it comes to combined PTT and GT nanosystems, photothermal‐sensitive NO donors are extensively explored as they enable both therapeutic functions to be achieved through a single NIR light irradiation.^[^ ^76^, ^83^ ^]^ Various types of thermal‐sensitive NO donors, including S‐nitrosothiols (RSNOs), bis‐*N*‐nitroso compounds (BNN), and *N*‐diazeniumdiolates (NONOates), have been explored for their ability to release NO gas under heat stimulation, making them suitable for integration with PTAs to achieve photothermal‐induced NO production.^[^ ^75^, ^84^ ^]^ RSNO, a biocompatible NO donor, is synthesized by reacting a thiol group (−SH) with *t*‐butyl nitrite (TBN).^[^ ^85^, ^86^ ^]^ Upon NIR laser irradiation, the PTAs generate robust photothermal effects that cleave the S─NO bonds in RSNO, leading to the release of NO gas.^[^ ^87^ ^]^ BNN derivatives, another type of high‐performance caged NO donor, have also been integrated with PTAs in recent years to realize controlled NO production.^[^ ^88^, ^89^, ^90^ ^]^ NONOates, a traditional type of NO donor, are formed through reactions between primary or secondary amines and NO gas under high pressure, base, and low‐temperature conditions.^[^ ^91^, ^92^ ^]^ The NO release from NONOates can be achieved through photothermal‐mediated homolytic scission of N─NO bonds.^[^ ^43^, ^93^ ^]^ Notably, photoacoustic (PA) imaging is often accompanied by PTAs, which combine the advantages of both optical imaging and ultrasound imaging. Besides, various inorganic PTAs and organic PTAs may also bring other imaging modalities like magnetic resonance (MR) imaging, computerized tomography (CT) imaging and fluorescence imaging, and so forth. This integration is expected to improve the accuracy of treatment, enabling better characterization and visualization of biological structures or pathological conditions.

Typically, PTAs are divided into two categories, that is, inorganic and organic PTAs. Inorganic PTAs present a myriad of advantageous attributes, encompassing robust light absorption, elevated photothermal conversion efficiency, adjustable optical properties, remarkable stability, biocompatibility, multifunctionality, compatibility with imaging modalities, and the potential for surface functionalization. These distinctive features render them immensely promising for diverse biomedical applications.^[^ ^22^, ^94^ ^]^ In addition to widely investigated noble metal nanomaterials (such as golden nanorods^[^ ^95^ ^]^) and metal sulfides (such as copper sulfide (CuS)^[^ ^96^ ^]^ and bismuth sulfide^[^ ^97^ ^]^), MXenes with distinctive 2D nanosheet structures and strong NIR light absorption have also been regarded as promising candidates for cancer nanotheranostics.^[^ ^98^, ^99^ ^]^

**FIGURE 1:**
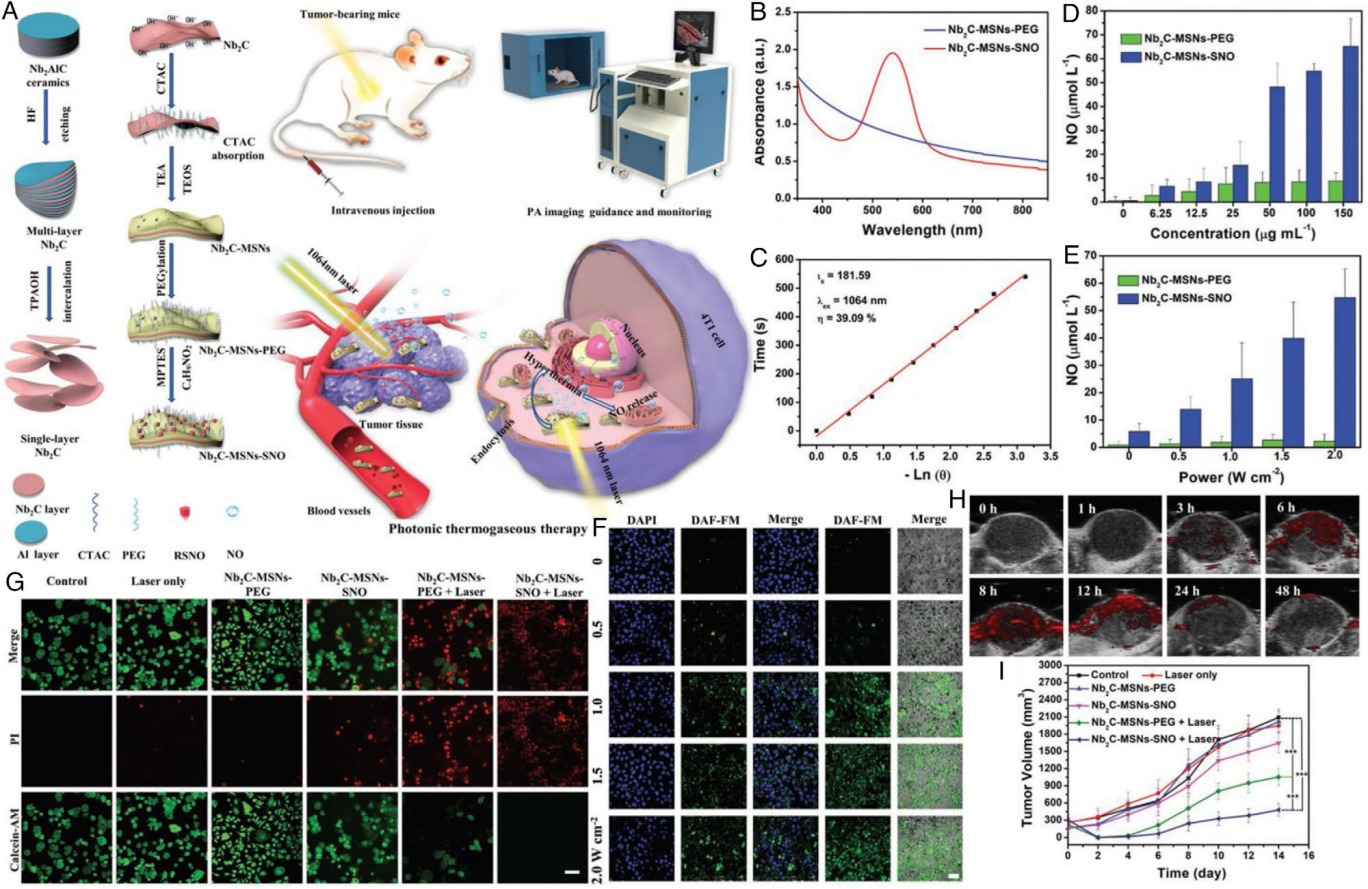
(A) Schematic illustration of the synthetic process of Nb_2_C‐MSNs‐SNO and photonic thermogaseous therapy against cancer with potential PA imaging guidance and monitoring. (B) UV–vis–NIR spectra of Nb_2_C‐MSNs‐PEG and Nb_2_C‐MSNs‐SNO after added into the Griess agents. (C) Time constant for heat transfer from the system by using the linear time data from the cooling period versus. Quantitative assessment of NO release from Nb_2_C‐MSNs‐SNO and Nb_2_C‐MSNs‐PEG under varied treatments including: (D) different power densities (0, 0.5, 1.0, 1.5, and 2.0 W cm^−1^, [Nb] = 50 µg mL^−1^) and (E) varied concentrations (1.5 W cm^−2^, [Nb] = 0, 6.25, 12.5, 25, 50, 100, and 150 µg mL^−1^). (F) Intracellular detection of NO release in 4T1 cancer cells after the coincubation with Nb_2_C‐MSNs‐SNO followed by laser irradiation at different power densities (0, 0.5, 1.0, 1.5, and 2.0 W cm^−2^, [Nb] = 50 µg mL^−1^; scale bar: 50 µm). (G) CLSM images of 4T1 cells stained by calcein AM (green) and PI (red) after different treatments. The green and red fluorescence represents live and dead cells, respectively. Scale bar: 50 µm. (H) In vivo PA images of tumor after intravenous administration of Nb_2_C‐MSNs‐SNO at different time intervals (0, 1, 3, 6, 8, 12, 24, and 48 h). (I) Time‐dependent tumor‐growth profiles after various treatments, including control, 1064 nm laser only, Nb_2_C‐MSNs‐PEG, Nb_2_C‐MSNs‐SNO, Nb_2_C‐MSNs‐PEG + 1064 nm laser, and Nb_2_C‐MSN‐SNO + 1064 nm laser. (*p*‐values: *p < 0.05, **p < 0.01, and ***p < 0.001). Reproduced with permission.^[^ ^51^ ^]^ Copyright 2019, Wiley‐VCH GmbH.

Recently, Yin et al. constructed a 2D Nb_2_C MXene nanosheet‐based multifunctional mesoporous composite for NIR‐II laser‐controlled NO generation, aiming at achieving PA imaging‐guided synergistic PTT/GT (Figure 1A).^[^ ^51^ ^]^ Briefly, the Nb_2_C MXene nanosheet was first synthesized and coated with a mesoporous silica layer (Nb_2_C‐MSNs), followed by surface modification to obtain Nb_2_C‐MSNs‐SNO. As shown in Figure 1B, the typical absorption peak around 540 nm demonstrated the successful loading of NO donor, while the decent absorbance at NIR region indicated the potential of Nb_2_C MXene nanosheet as PTA. Such Nb_2_C‐MSNs‐SNO composite nanosheets exhibited good photothermal performance and photostability, the PCE of which was calculated to be 39.09% under 1064 nm laser irradiation (Figure 1C). As expected, the thermal shock could sequentially trigger controllable generation of NO by breaking the S─NO bonds, showing concentration‐ and laser power‐dependent manners (Figure 1D,E). Specifically, about 55 µmol L^−1^ of NO was released after irradiation (2 W cm^−2^, 10 min) when [Nb] was 50 µg mL^−1^. Moreover, the strengthened green fluorescence in 4T1 cancer cells also evidenced PTT‐driven NO production (Figure 1F). This synergistic photonic thermogaseous therapy demonstrated an extremely enhanced cancer cell killing effect, and nearly no cells survived when the laser power was 1.5 W cm^−2^ (Figure 1G). Notably, the Nb_2_C‐MSNs‐SNO could also serve as a decent PA imaging contrast agent to guide the treatment process. The intensive PA signal at 8 h post‐injection verified the considerable tumor accumulation of Nb_2_C‐MSNs‐SNO (Figure 1H). In vivo therapeutic experiments displayed similar trends; the Nb_2_C‐MSNs‐SNO + Laser group exhibited the highest tumor inhibition rate in comparison to the other groups (Figure 1I). By integrating the features of each component, these multifunctional 2D composite nanosheets are anticipated to offer a new cancer therapeutic modality.

On the other hand, organic dyes have garnered significant attention in the field of biomedicine due to their rapid metabolism, resulting in minimal retention time and swift excretion from the body. However, when administered as small molecules, organic dyes often exhibit non‐specific biodistribution, leading to inadequate in vivo imaging efficiency. To address this limitation, researchers have explored the utilization of various nanocarriers for targeted delivery of organic dyes to the tumor site.^[^ ^100^, ^101^ ^]^ The aza‐boron‐dipyrromethene (aza‐BODIPY) core, which refers to the azabora‐dipyrromethene structure, has emerged as a highly valuable electron‐deficient motif in the development of NIR dyes. By adhering to specific design principles, aza‐BODIPY‐based compounds hold immense potential as PTAs for effective tumor treatments.^[^ ^102^, ^103^ ^]^

**FIGURE 2:**
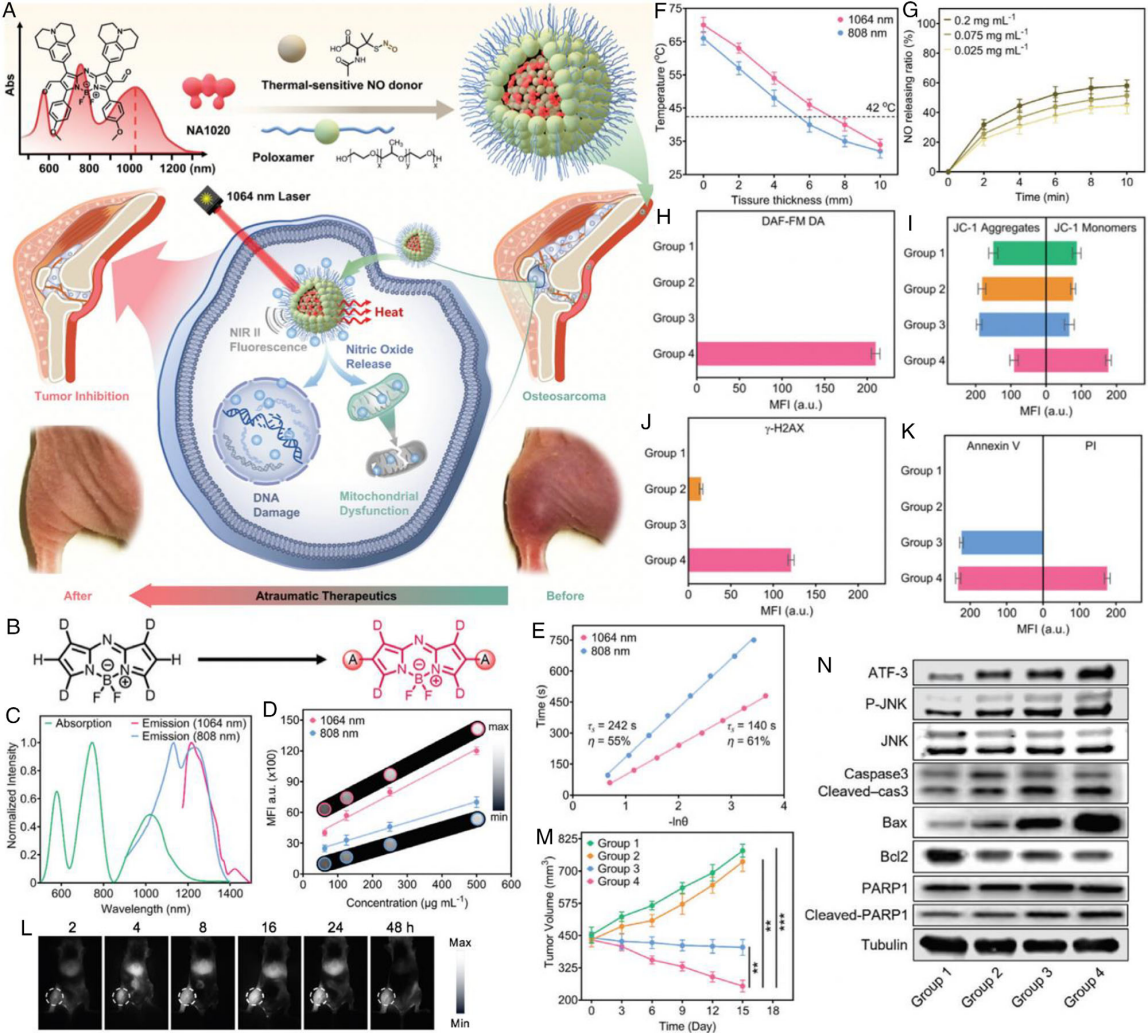
(A) Schematic of the gas/phototheranostic nanocomposite (NA1020‐NO@PLX). An enhanced ICT mechanism to introduced to develop the NIR‐II‐peak absorbing PTA of NA1020, which was combined with thermal‐sensitive NO donors to facilitate a combined low temperature PTT with GT. NA1020‐NO@PLX emitted the NIR‐II fluorescence to guide the heat generation that simultaneously activated NO release using laser at 1064 nm for atraumatic osteosarcoma therapy. (B) The illustrated enhanced ICT mechanism in the aza‐BODIPY modified with the electron withdrawing moieties on the 2, 6‐positions. (C) Absorption and emission (808, 1064 nm) spectra of NA1020@PLX. (D) Mean fluorescence intensities (MFI) of NA1020@PLX at different concentrations and the corresponding fluorescence images (𝜆_ex_ = 808, 1064 nm). (E) The time constant (τ_s_) and PCE under laser irradiation at 808 and 1064 nm. (F) Temperature variation curve of NA1020@PLX (200 µg mL^−1^) after 10 min irradiation (𝜆_ex_ = 1064 nm, *P* = 0.5 W cm^−2^) with the blocking of the chicken breast muscle (0–10 mm in sickness). (G) The NO releasing ratio from SNAP@PLX (1000 µg mL^−1^) at different temperatures in a water bath. (H) The quantified fluorescence intensity of the DAF‐FM DA channel. (I) Quantified fluorescence intensities in JC‐1 monomers and aggregates channels. (J) Quantified fluorescence intensities in 𝛾‐H2AX channel. (K) Quantified fluorescence intensities in Annexin V and PI channels. (L) The whole‐body NIR‐II imaging of 143B‐tumor‐bearing mice with NA1020@PLX via intravenous injection (𝜆_ex_ = 1064 nm). (M) Orthotopic 143B tumor volume variation curves in the four treatment groups. *n* = 5 biologically independent animals. (N) Tumor weights in the four treatment groups after the therapeutics. *n* = 5 biologically independent animals. Reproduced with permission.^[^ ^104^ ^]^ Copyright 2023, Wiley‐VCH GmbH.

For example, a gas/phototheranostic nanocomposite called NA1020‐NO@PLX was developed by Fang et al. for NIR‐II fluorescence imaging‐guided low temperature NIR‐II PTT/GT (Figure 2A).^[^ ^104^ ^]^ The NIR‐II peak absorbance was achieved by engineering an enhanced intramolecular charge transfer (ICT) mechanism on the template aza‐BODIPY NJ1060. As shown in Figure 2B,C, the modification of NJ1060 with electron‐withdrawing moieties at the 2, 6‐positions resulted in the construction of aldehyde‐modified NA1020, which exhibited a red‐shifted peak absorption reaching the NIR‐II region at 1020 nm. NA1020 with the amphiphilic poloxamer F127 (PLX) coated (NA1020@PLX) demonstrated the ability to emit NIR‐II fluorescence and generate heat with a high photothermal conversion efficiency (PCE) of 61% under low‐energy 1064 nm laser irradiation (Figure 2D,E). Even in the presence of thick chicken breast muscle, NA1020@PLX showed effective heat generation without causing significant tissue damage, indicating its potential for atraumatic therapy (Figure 2F). To further enhance the therapeutic efficacy, NA1020 was integrated with the thermal‐sensitive NO donor (S‐nitroso‐*N*‐acetylpenicillamine, SNAP) and then encapsulated in PLX to obtain NA1020‐NO@PLX. Under laser irradiation at 1064 nm, NA1020‐NO@PLX underwent a low‐temperature PTT process to break the S─NO bonds. Despite the wide range of concentrations of PTA employed, the rates of photothermal‐induced NO release showed minimal variations (ranging from 45% to 58%), highlighting the practical feasibility of the proposed gas/phototheranostics approach (Figure 2G). The presence of NO triggered additional mitochondrial dysfunction and DNA damage, enhancing tumor cell apoptosis and increasing the efficacy of PTT (Figure 2H–K). Moreover, the emitted NIR‐II fluorescence from NA1020‐NO@PLX allowed for precise tumor localization, enabling deep‐tissue orthotopic osteosarcoma therapy (Figure 2L). Thus, the tumor volumes in the NA1020‐NO@PLX + Laser group showed a steady decrease owing to the NO‐mediated low‐temperature PTT approach (Figure 2M). Accordingly, the expression of c‐Jun Nterminal kinase (JNK) was upregulated due to the presence of NO, stimulating the apoptosis‐specific genes expression via target transcription factors (TF). Moreover, JNK could also inhibit B‐cell lymphoma‐2 (Bcl2) and promote B‐cell lymphoma‐2‐associated X protein (Bax) release from the inner mitochondrial membrane via direct phosphorylation. The enhanced phosphorylated JNK (p‐JNK) by PTT/GT and activating transcription factor 3 (ATF3) facilitated the increase of Bax/Bcl2 ratio, which was associated with the increased expression of cleaved Caspase 3 and poly ADP‐ribose polymerase (PARP1) (Figure 2N). These enhanced apoptosis mechanisms highlight the potential clinical applications of such a gas/phototheranostic nanocomposite for deep‐tissue tumor treatment.

NIR‐absorbing semiconducting polymer nanoparticles (SPNs), known as PFTDPP, have also been reported to incorporate SNAP to create a core–shell nanotheranostic system (named PFTDPP‐SNAP), followed by a Pluronic F127 coating to ensure biocompatibility. The key of the design was PFTDPP, which was synthesized by copolymerizing diketopyrrolopyrrole (DPP) (a strong electrophilic molecule) with fluorene and thiophene (strong electron‐donating substitutes). This polymer composition enabled the SPNs to exhibit favorable heat generation for PTT and thermal‐triggered NO release for GT upon laser irradiation (PCE = 48% at 808 nm), along with dual‐mode NIR II fluorescence/PA imaging signals for precise tumor site location.^[^ ^105^ ^]^

Mesoporous polydopamine (M‐PDA), with exceptional biocompatibility and robust photothermal effect, also offers a high loading capacity for NO donor (*N,N*′‐di‐*sec*‐butyl‐*N,N*′‐dinitroso‐1,4‐phenylenediamine, BNN6). To further enhance the stability and performance of the delivery system, Wu et al. employed red blood cell membranes (RBCm) as the outer shell to create RBCm‐cloaked biomimetic delivery systems. Upon exposure to an 808 nm laser, the nanoerythrocytes induced hyperthermia to perform effective PTT (PCE = 23.8%) and simultaneously activate the release of NO by breaking the N─NO bonds within the tumor. Besides, the released NO and superoxide (O_2_^−•^) could generate reactive nitrogen species (ONOO^−^), a highly toxic species that enhanced the therapeutic effects for cancer treatment. More importantly, NO and ONOO^−^ inhibited the expression of hypoxia‐inducible factor‐1α (HIF‐1α) to alleviate tumor hypoxia, which further enhanced the therapeutic efficacy.^[^ ^106^ ^]^

### 2.2 Light‐sensitive NO donors for dual‐modal therapy

Light‐sensitive NO donors are a class of compounds that exhibit the capability to release NO upon exposure to light. These compounds have generated considerable interest in the field of cancer therapy due to their ability to provide precise spatial and temporal control over NO release. This feature is crucial for achieving targeted and controlled therapeutic interventions.^[^ ^43^, ^74^, ^107^ ^]^ While Section 2.1 discussed NIR light‐induced photothermal‐driven NO‐generating systems, it is also important to highlight other light‐sensitive NO donors that do not involve heat production. These alternative light‐sensitive NO donors offer unique advantages and contribute to the diverse toolbox of NO‐based therapeutic strategies.

**FIGURE 3:**
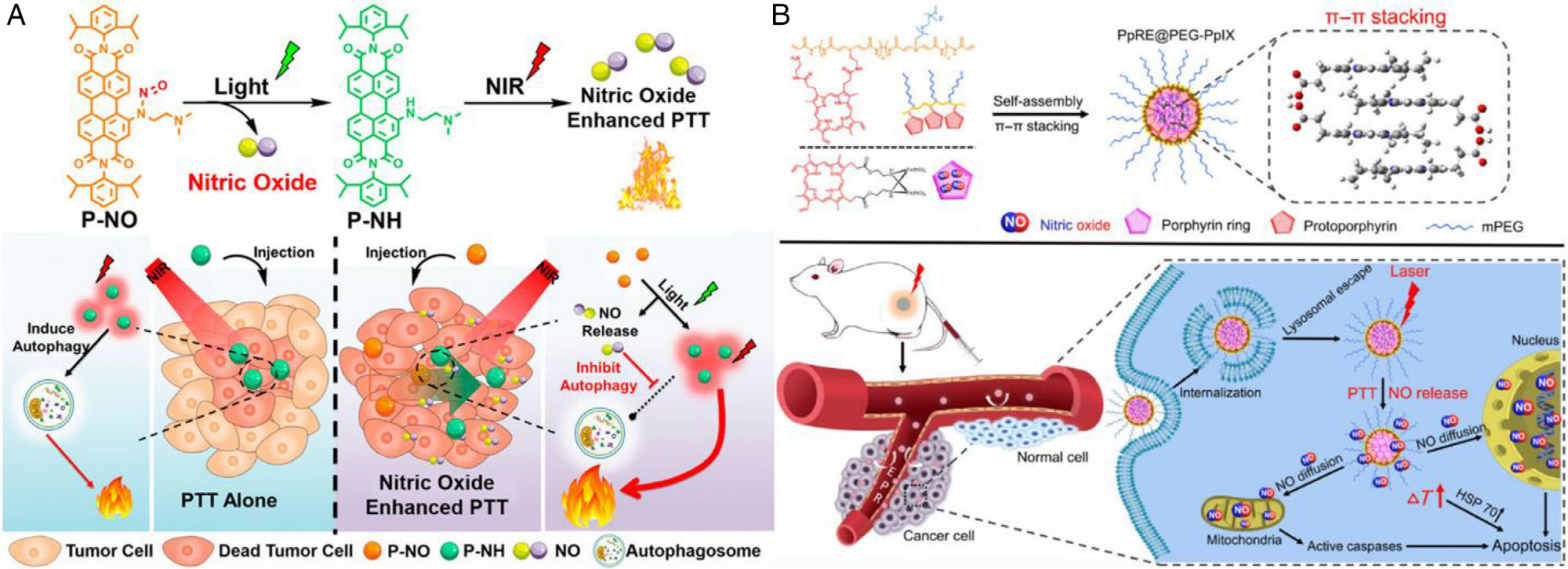
(A) Schematic illustration of dual‐light activatable P‐NO and mechanism of NO‐enhanced PTT in cancer therapy. Reproduced with permission.^[^ ^108^ ^]^ Copyright 2023, American Chemical Society. (B) Fabrication of PpRE@PEG‐PpIX and schematic illustration of PpRE@PEG‐PpIX for synergistic combination of PTT and NO for cancer treatment. Reproduced with permission.^[^ ^109^ ^]^ Copyright 2019, Tsinghua University Press.

For example, Ji et al. presented a dual‐light activatable perylenediimide derivative (P‐NO) that incorporated light‐responsive N‐nitrosamines onto a NIR PTA (P‐NH) for NO‐improved PTT (Figure 3A).^[^ ^108^ ^]^ As for better biomedical applications, the as‐synthesized P‐NO was transformed into nanoparticles in aqueous solutions, which could undergo a two‐step light‐activation process to enhance therapeutic efficacy. In the first step, upon irradiation with green light (520 nm), P‐NO released NO and transformed into P‐NH. This process involved the conversion of electron‐withdrawing group N‐nitrosamines into an electron‐donating NH group with NO simultaneously generated, and a 40 µm concentration of P‐NO was capable of releasing 33.6 µm NO. Subsequently, P‐NH induced direct cellular damage through photothermal ablation under NIR light irradiation (660 nm), while the release of NO from P‐NO inhibited the cell autophagy caused by PTT alone, significantly improving the cancer cell‐killing efficiency. In a mouse subcutaneous tumor model, the dual‐light activated PTT/GT using P‐NO nanoparticles resulted in significant tumor elimination. This approach offers a promising and straightforward method to construct activatable perylenediimide‐based PTAs for enhanced cancer treatment.

Huang et al. proposed an intelligent polymer nanoplatform based on hydrophobic protoporphyrin (PpIX) branches and porphyrin‐modified Roussin's red‐salt ester (PpRE), which were arranged through π–π stacking between two porphyrin rings.^[^ ^109^ ^]^ While Roussin's red salt (RRS) is a water‐soluble NO donor, whose main light absorption wavelength is in the UV range. The PpRE@PEG‐PpIX nanoparticles displayed a widened Soret band around 400 nm, indicating the presence of π–π stacking forces. Additionally, the Q band of the nanoparticles still showed an absorption peak at ≈637 nm, suggesting that the PpRE@PEG‐PpIX could be stimulated with a 637 nm laser for both PTT and GT. Notably, the nanoparticle did not require long‐time illumination (only need few seconds) to stimulate the production of NO. Therefore, the NO release principle of PpRE@PEG‐PpIX was presumed that PpIX ring directly absorbed light energy and transferred it to the nitrosyl group to release NO, with the advantage of high sensitivity for light‐controlled release behavior. This indicates the potential of the nanoparticles to selectively release NO as a therapeutic mechanism before inducing photothermal effects (Figure 3B).

### 2.3 Ultrasound‐sensitive NO donors for dual‐modal therapy

Ultrasound‐sensitive NO donors have caught attention in cancer therapy owing to the non‐invasive nature of ultrasound and its ability to penetrate deep into tissues. These compounds offer the potential for spatial and temporal control over NO release, allowing for targeted and controlled therapeutic interventions.^[^ ^71^, ^110^ ^]^

**FIGURE 4:**
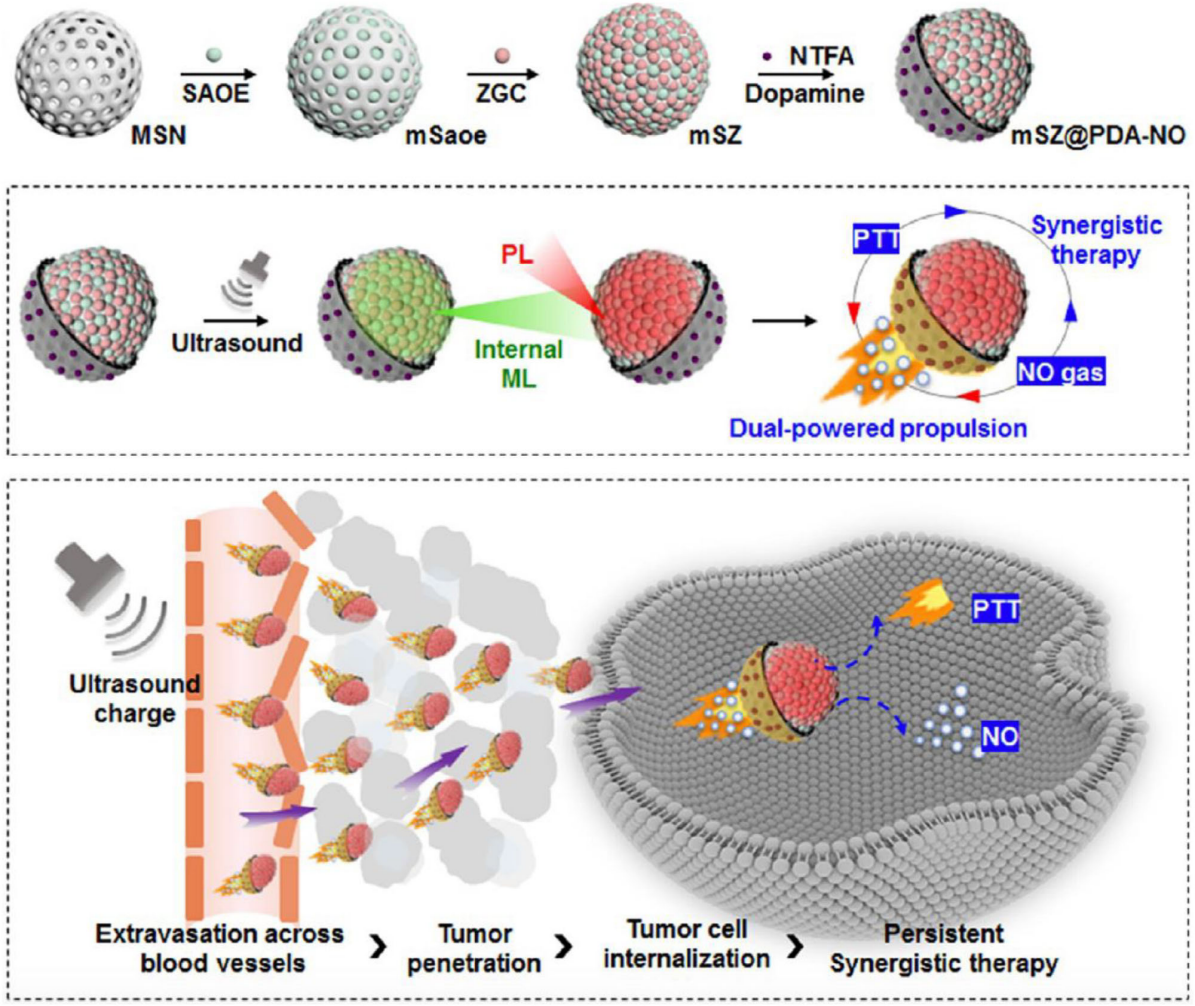
First, SAOE nanodots are deposited on MSNs to prepare mSaoe, followed by deposition of ZGC nanodots to obtain mSZ. PDA layers with loaded NO donors were selectively capped on one side of mSZ to construct Janus mSZ@PDA‐NO. Second, ultrasonication of the nanoplatform generates ML green emission from SAOE nanodots, which excite ZGC nanodots to emit PL of NIR. Third, the internal NIR excitation of PDA caps produces a persistent self‐thermophoretic force to propel NP motion, which promotes tumor accumulation, intratumoral penetration, and intracellular uptake of nanoplatform. In the meantime, NIR‐stimulated PTT and PL‐triggered NO release achieve a synergistic antitumor efficacy. Reproduced with permission.^[^ ^111^ ^]^ Copyright 2023, American Chemical Society.

In a recent study, Zhang et al. constructed ultrasound‐chargeable persistent luminescence (PL) nanoparticles for cancer PTT, concurrent thermophoresis‐propelled motion, and NO release (Figure 4).^[^ ^111^ ^]^ The proposed strategy involved the use of PL nanodots of SrAl_2_O_4_:Eu^2+^ (SAOE) and ZnGa_2_O_4_:Cr^3+^ (ZGC), which were deposited on mesoporous silicates to create mSZ nanoparticles. The mSZ were then partially coated with PDA caps and loaded with NO donor (4‐nitro‐3‐(trifluoromethyl)aniline, NTFA), resulting in Janus mSZ@PDA‐NO. Ultrasonication of the mSZ generated PL emissions, serving as an internal NIR source that continuously excited the PDA caps and induced a mild photothermal effect to heat tumors, as well as generating a thermophoretic force and NO gas propellers. Besides, these forces drove the motion of the mSZ@PDA‐NO within the tumor. In contrast to intermittent NIR illumination, the persistent motion of ultrasound‐activated nanoparticles enhanced cellular uptake and enabled long‐lasting PTT and intracellular NO levels, thus leading to significantly higher tumor growth inhibition, longer animal survival, and increased intratumoral NO levels. This approach addresses challenges associated with tumor cell accessibility and intermittent light excitation, offering potential improvements in cancer treatment.

### 2.4 ROS‐sensitive NO precursor for dual‐modal therapy

ROS, such as H_2_O_2_ and singlet oxygen (^1^O_2_), play a crucial role in oxidative stress. In the context of biomedical applications, ROS‐sensitive NO precursors have been designed to selectively release NO under conditions of oxidative stress. L‐arginine (L‐Arg), an amino acid, serves as the precursor for endogenous NO production in the body. In recent years, researchers have developed nanosystems capable of generating NO by incorporating L‐Arg or its derivatives.^[^ ^112^, ^113^, ^114^ ^]^ With cancer cells known to exhibit overproduction of H_2_O_2_, the conversion of L‐Arg to L‐citrulline can be leveraged to specifically generate NO for cancer therapy.^[^ ^115^, ^116^ ^]^

**FIGURE 5:**
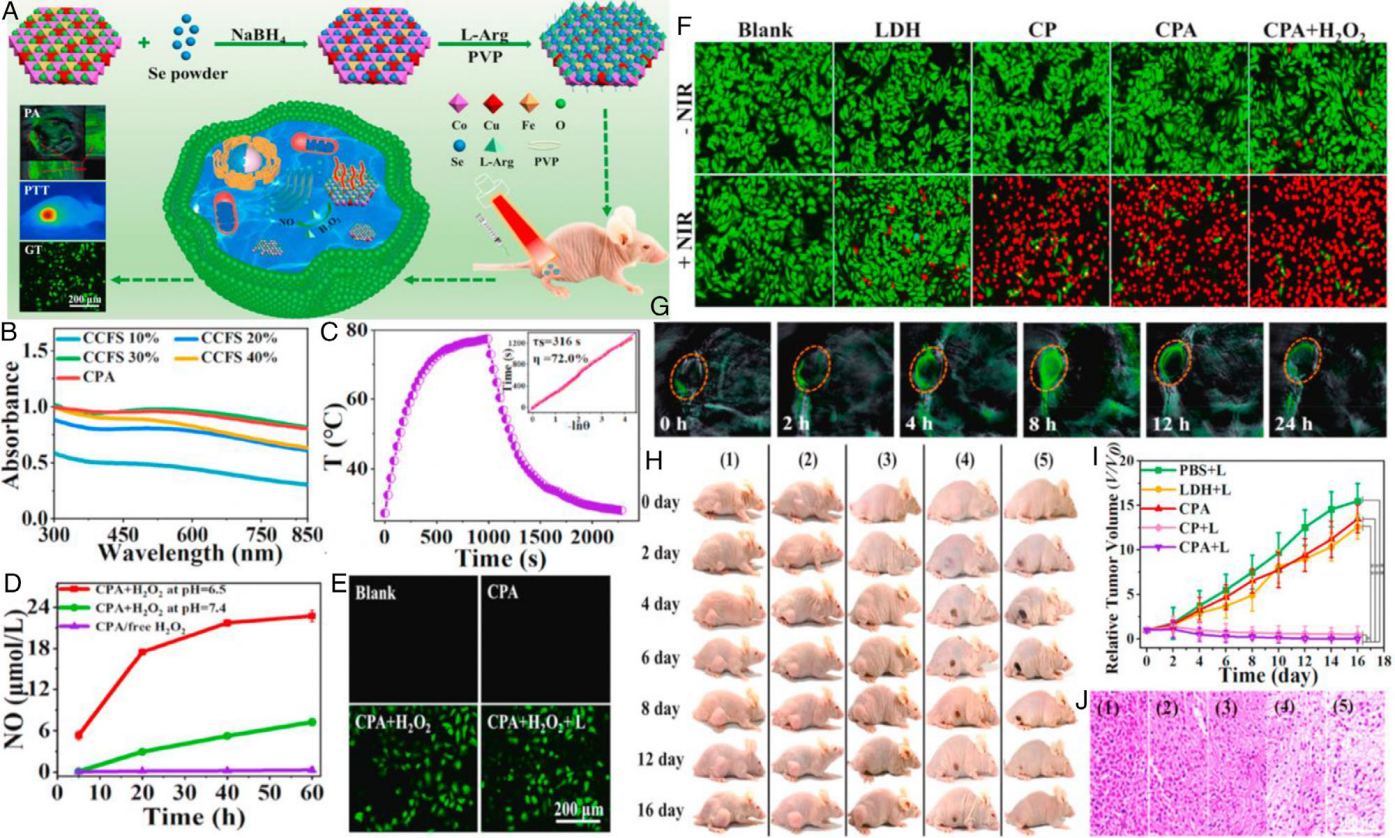
(A) A schematic illustration of the preparation of the CPA nanocomposite for PA image‐guided synergistic PTT/GT. (B) UV–vis absorption spectra of CCFS NSs with various Cu ratios and the CPA nanocomposite (30% Cu). (C) Calculation of photothermal conversion efficiency of CPA at pH 7.4. (D) The NO concentration generated by CPA with or without H_2_O_2_ under neutral (pH 7.4) or acidic (pH 6.5) conditions. Error bars denote ± s.d. (*n* = 3). (E) HepG2 cells stained with the NO fluorescence probe DAF‐FM DA after being treated with blank, CPA, CPA + H_2_O_2_, and CPA + H_2_O_2_ + laser. The green fluorescence intensity represents the NO level. (F) Calcein‐AM/PI staining images of HepG2 cells afte incubation with CoCuFe‐LDH, CPA + H_2_O_2_, CP and CPA at various concentrations (without or with 808 nm laser irradiation at a power density of 1.0 W cm^−2^ for 10 min). (G) In vivo PA imaging of the tumor (highlighted by a red circle) at various time points after a mouse was intravenously injected with CPA (dose = 10 mg kg^−1^). (H) Digital photographs of mice recorded every other day over 16 days after various treatments. (I) Tumor growth curves (**p < 0.01). (J) Tumor tissue slices stained with H&E after 16 days of treatment. Reproduced with permission.^[^ ^117^ ^]^ Copyright 2021, Elsevier Inc.

Recently, Wu et al. prepared an ultrathin Cu‐loaded CoCuFe‐selenide (CCFS) with polyvinyl pyrrolidone (PVP) and L‐Arg modified (denoted as CPA) for PA imaging‐guided synergistic PTT/GT (Figure 5A).^[^ ^117^ ^]^ Interestingly, it was found that the Cu‐doped ratio significantly impacted the PTT performance of CCFS. As shown in Figure 5B, the 30% Cu‐doped group exhibited the maximum light absorption at 808 nm, which was selected to construct the CPA nanocomposite. After 10 min of laser irradiation, the CPA dispersion displayed a temperature elevation of 45.9°C, and a superior PCE was calculated to be 72% (Figure 5C,D). Subsequently, the NO‐generating behavior of CPA was investigated with/without H_2_O_2_ treatment under different pH values. Since an acidic environment could improve the oxidation performance of H_2_O_2_, more L‐Arg was consumed to produce NO at pH 6.5 (3.4‐fold higher) in contrast to that at pH 7.4 (Figure 5E). Accordingly, the intracellular NO detection (green fluorescence) further demonstrated the essential role of H_2_O_2_ for L‐Arg oxidation (Figure 5F). Benefiting from the synergistic PTT/GT, only 8.2% of the HepG2 cells survived with a CPA concentration of 20 µg mL^−1^ (Figure 5G,H). Moreover, the obviously enhanced signal intensity verified the feasibility of CPA as a potent PA imaging contrast agent compared to LDH (Figure 5I,J). After injection for 8 h, the PA signals at the tumor site reached their peak, confirming the distinct accumulation of CPA (Figure 5K). In vivo experiments showed that tumors in CPA + NIR group were completely ablated due to combined antitumor effects. Meanwhile, a rapid wound healing process was noticed as NO could promote the production of various growth factors (Figure 5L–N).

### 2.5 GSH‐sensitive NO donors for dual‐modal therapy

GSH is a small molecule that plays a vital role in maintaining the redox balance within cells.^[^ ^118^ ^]^ GSH‐sensitive NO donors have garnered interest in the field of cancer therapy due to their ability to selectively release NO in tumor cells. Tumor cells often exhibit elevated levels of GSH compared to normal cells, which makes GSH a suitable trigger for targeted NO release with minimized off‐target effects.^[^ ^119^, ^120^ ^]^

For example, Li et al. engineered a theranostic agent (named CP‐bF@PEG) for high‐contrast cancer NIR‐II fluorescence imaging‐guided precision PTT/GT.^[^ ^121^ ^]^ CP‐bF@PEG was composed of a NIR‐II‐absorbing CP (PTTBBT) and a NO donor (benzofuroxan) with pegylated Pluronic F127 coating. The loaded benzofuroxan could release NO in a controlled manner triggered by a high concentration of GSH, in which the nucleophilic thiol group was added to the conjugated structure of furoxan to substitute NO. In detail, the released NO reached a plateau under 3 mm GSH activation with a yield of 81% and this NO generation lasted for 120 min. Simultaneously, PTTBBT, the NIR‐II‐absorbing component, was able to absorb laser energy in the NIR‐II range (1064 nm) to generate both NIR‐II fluorescence and heat. This property allowed for excellent PTT in deep tissue‐penetration and high‐contrast imaging with reduced background biological fluorescence. The theranostic agent demonstrated pronounced tumor therapeutic efficacy while minimizing side effects. This strategy has immense potential for the development of a new generation of nanomedicines for effective and targeted cancer treatment.

## 3 PTT/GT‐BASED TRIPLE‐MODAL THERAPY

Tumors exhibit notable diversity, complexity, and heterogeneity, posing significant challenges for effective treatment, even with dual‐modal therapies.^[^ ^122^, ^123^ ^]^ To confront these challenges, researchers have actively pursued the integration of multi‐modal therapeutic components within a single nanoplatform, employing rational design strategies. The ultimate objective of this approach is to achieve super‐additive anticancer efficacy and overcome the limitations associated with individual modalities.^[^ ^124^, ^125^, ^126^, ^127^, ^128^ ^]^ Within this section, we provide a summary of several triple‐modal treatments, encompassing combinations such as PTT/GT/CHT, PTT/GT/PDT, PTT/GT/ART, PTT/GT/RT, and PTT/GT/IT.

### 3.1 PTT/GT/CHT

CHT has proven to be indispensable in clinical cancer treatment, and researchers have explored diverse small‐molecule drugs over the past few decades.^[^ ^129^, ^130^, ^131^ ^]^ Despite efforts to enhance the efficacy of chemotherapy and minimize side effects through intelligent drug delivery systems, the emergence of multidrug resistance (MDR) poses a significant challenge to successful CHT.^[^ ^132^, ^133^, ^134^, ^135^ ^]^ Recent studies have revealed the potential of NO as a promising strategy to overcome MDR by inhibiting the expression of P‐glycoprotein (P‐gp), a membrane transporter protein responsible for actively extruding chemotherapeutic drugs from cancer cells, thereby reducing their intracellular concentration and effectiveness.^[^ ^136^, ^137^ ^]^ Moreover, PTT can downregulate drug efflux pumps and increase drug retention in tumor cells, overcoming multidrug resistance. It is worth mentioning that both PTT and NO gas are capable of improving blood flow and vascular permeability, allowing better delivery of chemotherapeutic drugs to the tumor site.^[^ ^138^, ^139^ ^]^

**FIGURE 6:**
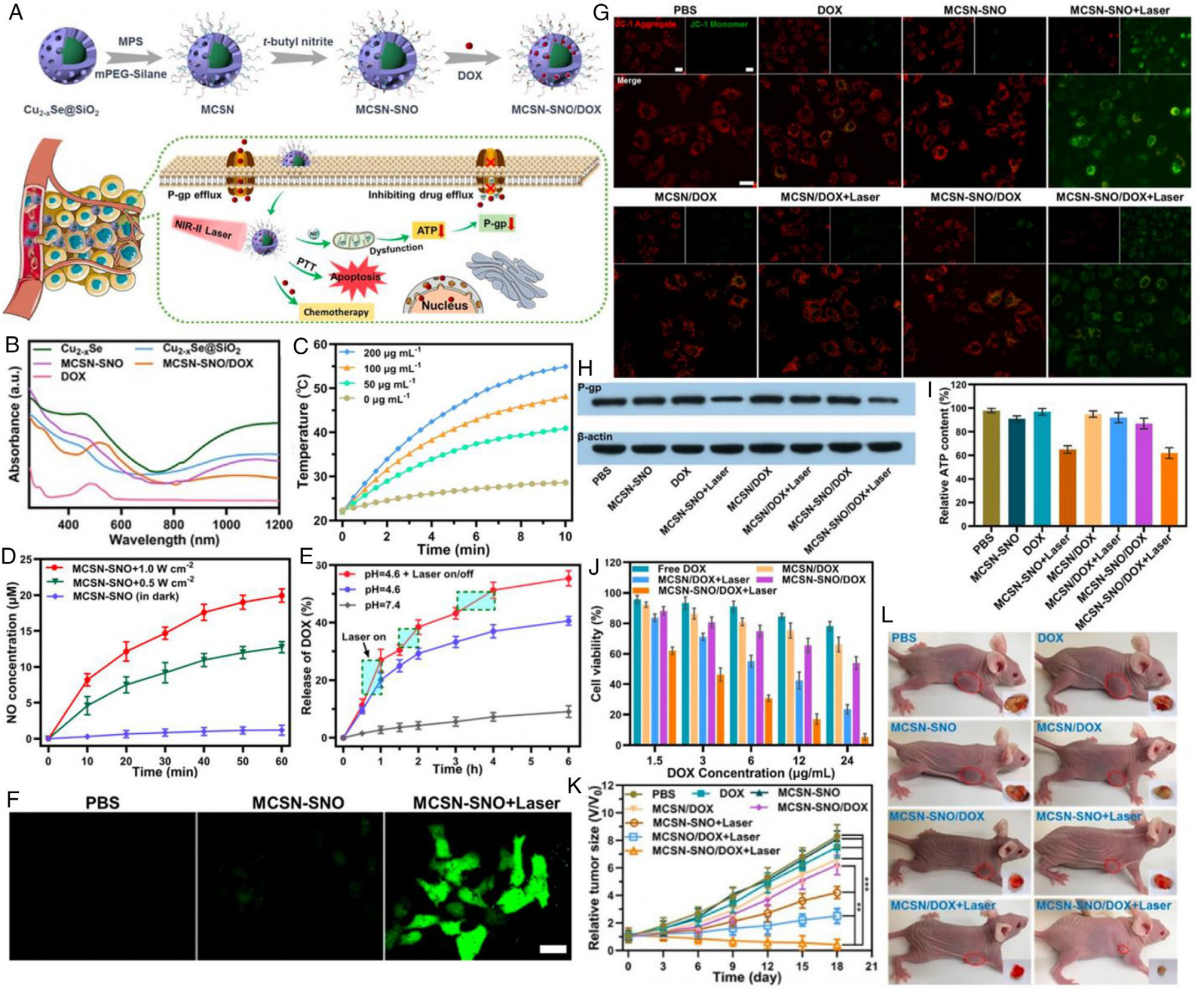
(A) Schematic illustration of the synthetic procedure of MCSN‐SNO/DOX and the process of photothermal‐responsive NO gas release for overcoming multidrug resistant tumor by combination therapy. (B) UV–vis–NIR absorbance spectra of DOX, Cu_2−_ *_x_* Se, Cu_2−_ *_x_* Se@SiO_2_, MCSN‐SNO and MCSN‐SNO/DOX. (C) The temperature elevation of the aqueous dispersion of MCSN‐SNO with different concentration. (D) Generation of NO gas from MCSN‐SNO in PBS solution with or without 1064 nm laser irradiation at power density of 0.5 and 1.0 W cm^−2^. (E) The release profile of DOX from MCSN‐SNO/DOX at pH = 7.4 or pH = 4.6 and the laser triggered release. (F) Fluorescence images of Hep‐G2/ADR cells after incubation for 4 h with MCSN‐SNO in the presence/absent of 1064 nm laser. Scale bar = 30 µm. (G) Fluorescence JC‐1 staining of Hep‐G2/ADR cells after receiving various treatments. (H) Western blot analysis of P‐gp in Hep‐G2/ADR cells after treatment with different groups. (I) Relative ATP content in the cells after different treatments. NIR laser: 1064 nm for 10 min, 1.0 W cm^−2^. Scale bar = 30 µm. (J) MTT assay for determining cytotoxicity of different treatments incubated with Hep‐G2/ADR. (K) The relative tumor volume change of each group of tumor‐bearing mice during therapy. (L) Photographs of mice and the solid tumors removed from different treatment groups at the termination of the study. Reproduced with permission.^[^ ^140^ ^]^ Copyright 2021, Elsevier Inc.

An intelligent triple‐modal therapeutic nanoplatform with the ability to against MDR was designed by Huang et al.^[^ ^140^ ^]^ Based on mesoporous core–shell structured nanocomposites (MCSN), Cu_2−_ *_x_* Se@SiO_2_, the nanoplatform was obtained after encapsulation of both NO donor (SNO) and doxorubicin (DOX) (Figure 6A). The UV–vis–NIR curve of as‐synthesized MCSN‐SNO/DOX not only presented the typical peak of DOX around 480 nm but also intensive NIR‐II absorption due to the presence of Cu_2−_ *_x_* Se (Figure 6B). After illumination by a 1064 nm laser for 10 min, the MCSN‐SNO aqueous dispersion (200 µg mL^−1^) showed a rapid temperature increase from 22 to 55°C, while only a slight elevation (Δ*T* = 6°C) was found in the control group (Figure 6C). Such remarkable hyperthermia from photothermal conversion could cause the breakage of S─NO bonds. Figure 6D illustrated that higher laser power density led to more NO production and 20 µm of NO was generated with 60 min (1064 nm, 1 W cm^−2^). More importantly, the laser irradiation promoted the DOX release and the accumulative release rate increased from 42% (pH = 4.6) to 55.4% when exposed to NIR‐II light (Figure 6E). Accordingly, the vivid green fluorescence in the MCSN‐SNO + Laser‐treated Hep‐G2/ADR cells indicated PTT‐induced NO production (Figure 6F). The generated NO gas was able to cause mitochondrial damage and adenosine triphosphate (ATP) decrease, resulting in the downregulation of P‐gp expression and an increase in intracellular DOX accumulation to overcome chemoresistance (Figure 6G–I). Specifically, free DOX‐ or MCSN/DOX‐treatment with only 22% or 34% cell death revealed the remarkable drug resistance of Hep‐G2/ADR cells, but about 95% of the Hep‐G2/ADR cells were killed in the MCSN‐SNO/DOX + Laser group due to the synergistic PTT/GT/CHT (Figure 6J). Similar trends were also found in the Hep‐G2/ADR tumor‐bearing nude mice single CHT induced moderate tumor growth inhibition (20.5% and 25.3% for MCSN/DOX and MCSN‐SNO/DOX, respectively), while dual‐modal PTT/GT and PTT/CHT suppressed tumor growth more effectively (49.4% and 69.9% for MCSN‐SNO + Laser and MCSN/DOX + Laser, respectively). Comparatively, tumor growth was almost completely inhibited after MCSN‐SNO/DOX + Laser treatment owing to the triple‐modal therapeutic effects (Figure 6K,L).

**FIGURE 7:**
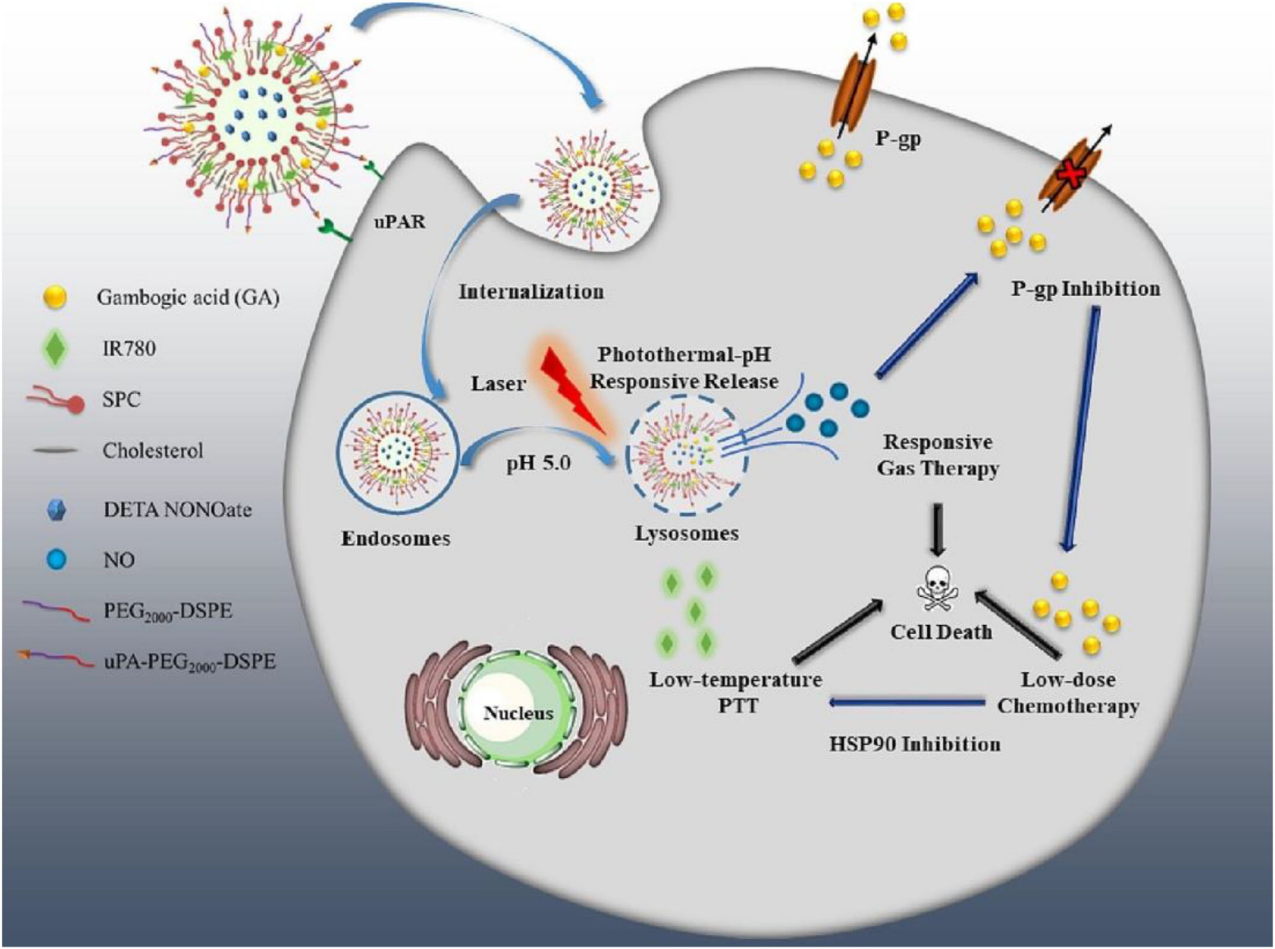
Schematic illustration of cascade amplification therapy of triple‐negative breast cancer mediated by a quad‐functional on‐demand released nanomissile (IGN Lipo). Reproduced with permission.^[^ ^141^ ^]^ Copyright 2023, Elsevier Inc.

Wang et al. constructed a quad‐functional on‐demand released nanomissile (IGN Lipo) that mainly contained IR‐780, gambogic acid (GA), diethylenetriamine diazeniumdiolate (DETA NONOate), PEG_2000_‐DSPE, and uPA‐PEG_2000_‐DSPE for cascade amplification treatment of triple‐negative breast cancer.^[^ ^141^ ^]^ The PEGylated and urokinase‐type plasminogen activator (uPA)‐modified nanomissile could enhance the circulation time in the body and increase drug accumulation in tumors. After being taken up by tumor cells, the loaded IR780 iodide (IR780) generated mild photothermal effects under NIR laser irradiation, which collaborated with the intracellular acidic environment to trigger the release of NO gas and GA. Among them, GA not only acted as a chemotherapeutic agent but also inhibited the activity of HSP90 to overcome photothermal resistance for enhanced low‐temperature PTT. NO gas contributed to GT as well as down‐regulating the expression of P‐gp to reverse MDR. Overall, this quad‐functional on‐demand released nanomissile achieves cascade‐amplified therapeutic effectiveness by suppressing tumor resistance proteins, showing promising potential for clinical applications in the treatment of triple‐negative breast cancer (Figure 7).

### 3.2 PTT/GT/PDT

As it is well known, ROS such as H_2_O_2_ and ^1^O_2_ have the ability to oxidize L‐Arg to generate NO.^[^ ^112^, ^142^ ^]^ PDT, another form of phototherapy, typically involves the use of lasers, photosensitizers, and molecular oxygen.^[^ ^143^, ^144^ ^]^ The production of ^1^O_2_ is a crucial aspect of the PDT process. Therefore, the incorporation of L‐Arg with various photosensitizers can offer distinct advantages in the context of synergistic GT/PDT. This innovative approach, employing light as an external stimulus, enables minimally invasive treatment, precise spatiotemporal selectivity, and reduced side effects.^[^ ^145^, ^146^, ^147^ ^]^ When PTT/NO integrated, tumor blood flow and oxygenation can be enhanced, leading to improved delivery and penetration of PSs as well as relieved tumor hypoxia. This is particularly beneficial for PDT, as the production of ROS in PDT is O_2_‐dependent.^[^ ^148^, ^149^ ^]^ Additionally, previous studies have indicated that NO can increase the sensitivity of cancer cells to ROS and can even react with ROS to form highly toxic ONOO^−^.^[^ ^150^ ^]^

**FIGURE 8:**
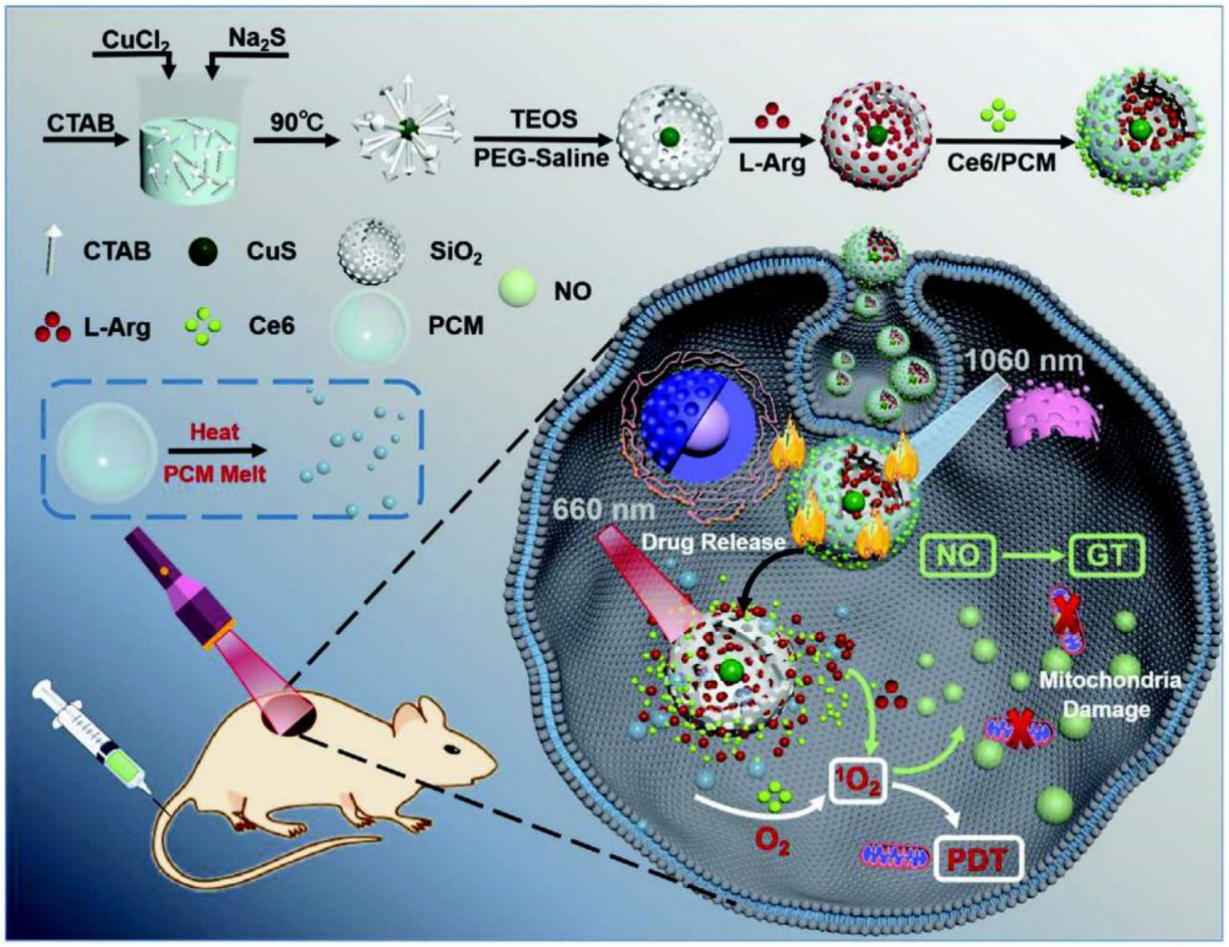
Schematic diagram of the CuS@SiO_2_‐L‐Arg@PCM‐Ce6 nanoparticles for thermal controlled drug release and synergistic photodynamic/gas therapy. Reproduced with permission.^[^ ^151^ ^]^ Copyright 2021, Wiley‐VCH GmbH.

For example, Zhu et al. proposed a “Linkage Mechanism” method using photothermal controllable multi‐shell nanoagent CuS@SiO_2_‐L‐Arg@PCM‐Ce6 (denoted as CSLPC) for combined PTT/GT/PDT (Figure 8).^[^ ^151^ ^]^ Briefly, the core‐shell CuS@SiO_2_ with typical porous structures was first synthesized, followed by L‐Arg loading and phase‐change material (PCM) coating, and chlorin e6 (Ce6) was finally encapsulated in the PCM. When exposed to a 1060 nm laser, the inner CuS nanoparticles could induce outstanding photothermal effects (PCE = 50.79%) that melted PCM to precisely trigger the release of both Ce6 and L‐Arg. In the presence of 660 nm laser illumination, the released Ce6 underwent the photodynamic process to generate abundant ^1^O_2_, which was able to oxidize the L‐Arg to produce NO. The “Linkage Mechanism” realized accurate cargo release within the photothermal stimulation at a specific tumor site, while the in situ chain reaction of PDT and GT was capable of avoiding premature gas leakage in normal tissues. In addition, CuS and Ce6 could also act as ideal fluorescence imaging and NIR‐II PA imaging contrast agents to guide the therapies. As expected, both in vitro and in vivo experiments evidenced the highly effective antitumor outcomes of CSLPC after 1060 and 660 nm laser concurrent treatment. The nanoagent offers a new concept for designing intelligent drug delivery systems as well as enlarging the synergetic treatment effects of multiple therapeutic paradigms.

In terms of laser setup, synchronization, and targeting accuracy, the utilization of two separate lasers for both PTT and PDT turns out to be really complex. Therefore, researchers have directed their efforts toward alternative strategies aimed at simplifying the process. One particularly popular approach is the utilization of a single laser to activate a dual‐functional agent that possesses both photothermal and photosensitizing properties. This approach offers several advantages in terms of streamlining the treatment procedure and enhancing overall efficiency.^[^ ^152^, ^153^, ^154^ ^]^

**FIGURE 9:**
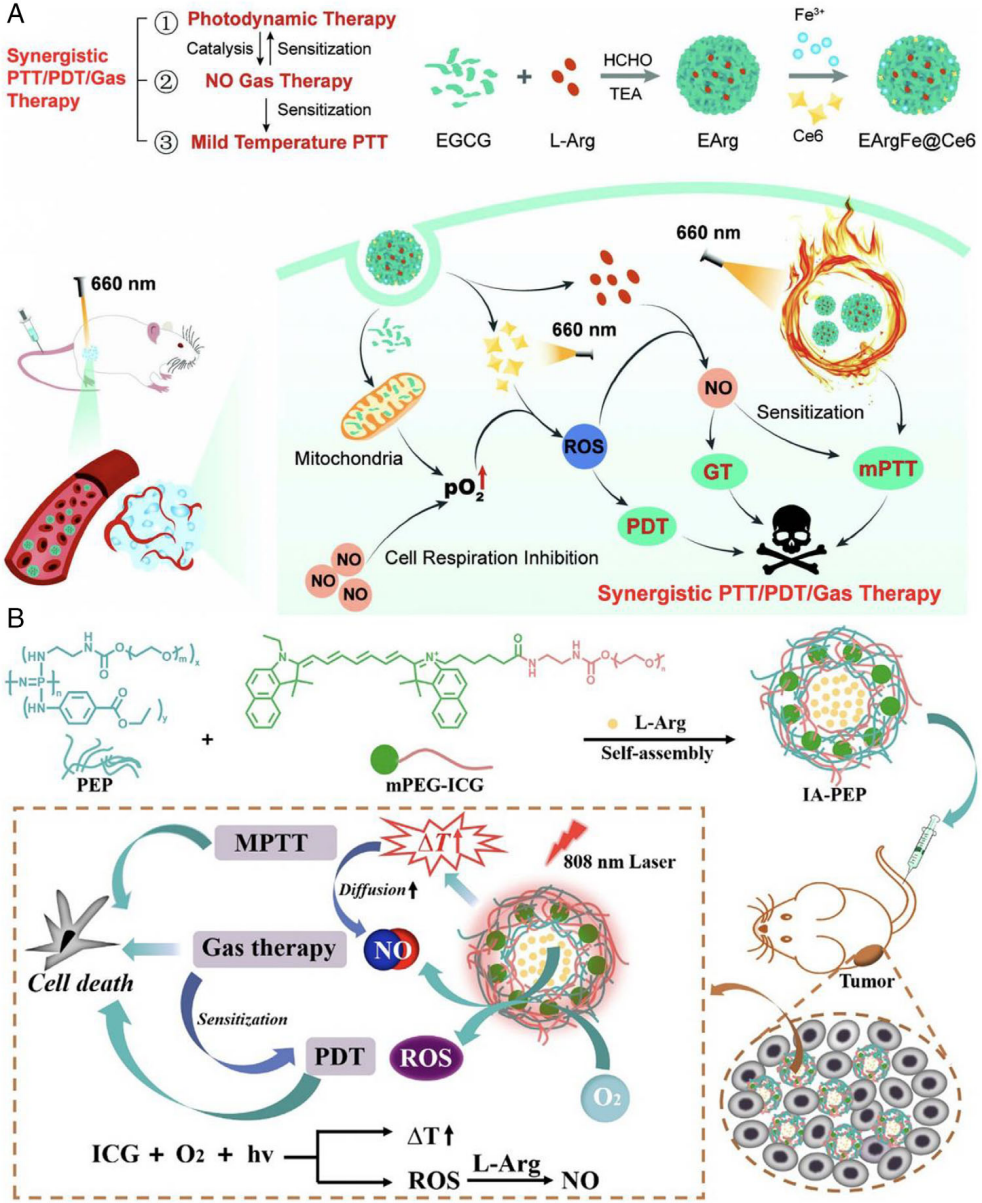
(A) Scheme illustration of the preparation process of EArgFe@Ce6 and the mechanism for 660 nm light‐triggered synergistic PTT/PDT/GT by EArgFe@Ce6. Reproduced with permission.^[^ ^155^ ^]^ Copyright 2023, Wiley‐VCH GmbH. (B) Schematic illustration of the NIR induced ternary synergistic cancer therapy based on L‐Arg and PEGylated ICG modified nanovesicles. Reproduced with permission.^[^ ^157^ ^]^ Copyright 2022, Elsevier Inc.

Shi et al. described a novel nanoplatform based on a chemical combination of epigallocatechin‐3‐gallate (EGCG) with L‐Arg (EGCG‐Arg, denoted as EArg).^[^ ^155^ ^]^ The further addition of Fe^3+^ ions to EArg allowed for coordination between EGCG and Fe^3+^, resulting in the formation of EArgFe with excellent photothermal capability (PCE = 41.7%). At last, Ce6 was physically adsorbed onto the surface to create EArgFe@Ce6. Due to the strong binding capability of EGCG to mitochondrial outer membrane proteins, EArgFe@Ce6 was endowed with mitochondria‐targeting properties. Upon 660 nm light irradiation, EArgFe@Ce6 generated massive ROS, which, in turn, catalyzed L‐Arg to produce a significant amount of NO. The localized generation of NO within the mitochondria further suppressed cell respiration, improving the PDT effect. Additionally, the release of NO helped sensitize PTT by sharing the 660 nm laser. The experimental results demonstrated that the synergistic PTT/GT/PDT was able to effectively ablate tumors in mice (Figure 9A).

Indocyanine green (ICG) is a NIR dye that has been widely used for both PTT and PDT alone or in combination due to its excellent light absorption and conversion properties.^[^ ^156^ ^]^ For example, Wang et al. developed a nano‐vesicle system called IA‐PEP, which integrated mPEG‐ICG and L‐Arg within the amphiphilic polymer (poly[(PEG)(ethyl‐*p*‐aminobenzoate)phosphazene], PEP) nano‐vesicles.^[^ ^157^ ^]^ The unique nanostructure of the vesicles enabled a high loading capacity for both mPEG‐ICG and L‐Arg, with loading contents of 15.9% and 17.95%, respectively. Importantly, the vesicles effectively prevented leakage of the cargoes. Under 808 nm laser irradiation, IA‐PEP produced ROS to perform PDT, and further reacted with L‐Arg to form NO for effective GT. Simultaneously, the mild heat (PCE = 15.93%) generated by IA‐PEP also contributed to the antitumor effect without causing significant damage. In vivo experiments conducted on nude mice with MCF‐7 tumor xenografts confirmed the potent antitumor efficacy of IA‐PEP under 808 nm laser irradiation, resulting in complete tumor elimination. Overall, the designed IA‐PEP nano‐vesicle system presents a promising treatment approach for synergistic cancer therapy by combining PDT, GT, and mild PTT (Figure 9B).

### 3.3 PTT/GT/ART

The curative outcome of traditional PDT is severely restricted by the hypoxic TME.^[^ ^158^, ^159^, ^160^ ^]^ Therefore, the development of O_2_‐independent PDT has become a hot research topic in recent years. Typically, the commonly used free radical initiator 2,2′‐azobis[2‐(2‐imidazolin‐2‐yl)propane]‐dihydrochloride (AIPH) can be bundled with PTAs and decomposed to generate alkyl radicals under NIR laser irradiation for O_2_‐independent PDT, also known as thermodynamic therapy or ART.^[^ ^161^, ^162^ ^]^ PTT and NO gas can improve tumor blood flow to enhance the delivery and penetration of free radical initiators, this improved distribution leads to more uniform and effective tumor cell destruction throughout the entire tumor volume. The three therapeutic functions can be performed under one single NIR laser irradiation at the tumor site, showing reduced off‐target effects on healthy cells and tissues.^[^ ^163^, ^164^ ^]^ Such a strategy allows for effective tumor cell destruction regardless of the oxygenation status, expanding the applicability of PDT to a wider range of tumor conditions.

For example, Wang et al. first synthesized mesoporous silica coated gold nanorods (AuNRs@MSN) with good photothermal effects, which served as a decent vehicle to separately deliver AIPH and SNO (A‐AuNRs@MSN‐SNO); then a supramolecular complex consisting of an amino pillar[5]arene (NP5) and a galactose derivative (G) was functionalized (A‐AuNRs@MSN‐SNO@NP5G) through host‐guest/electrostatic interactions to realize glyco‐targeting ability.^[^ ^165^ ^]^ The as‐prepared AuNRs@MSN was uniform and monodisperse, and further modification of NP5G was reflected in the denser layer. Compared with AuNRs, a red shift was found in the UV–vis curve of AuNRs@MSN, signifying its possibility for PTT. The NIR‐induced hyperthermia not only could promote the decomposition of AIPH to generate alkyl radicals even under hypoxic conditions but also trigger the release of NO. In contrast to HL‐7702 cells, the HepG2 cells incubated with the Rhodamine B‐labeled AuNRs@MSN‐SNO@NP5G showed stronger red fluorescence, evidencing that the glycol‐targeting capacity originated from the specific recognition between the galactose residue of G and the overexpressed ASGPR of HepG2 cells. The brightest green fluorescence in the A‐AuNRs@MSN@NP5G + NIR group further confirmed the NIR‐light‐driven alkyl radicals/NO‐generating abilities at the cellular level. Therefore, the A‐AuNRs@MSN@NP5G with NIR group possessed the most significant cell‐killing effects, and only 15.9% of HepG2 cells survived after incubation for 72 h. This work fabricates a new supramolecular hybrid nanoplatform for cancer‐targeted triple‐modal treatment, which may inspire further investigation into supramolecular nanomedicines.

**FIGURE 10:**
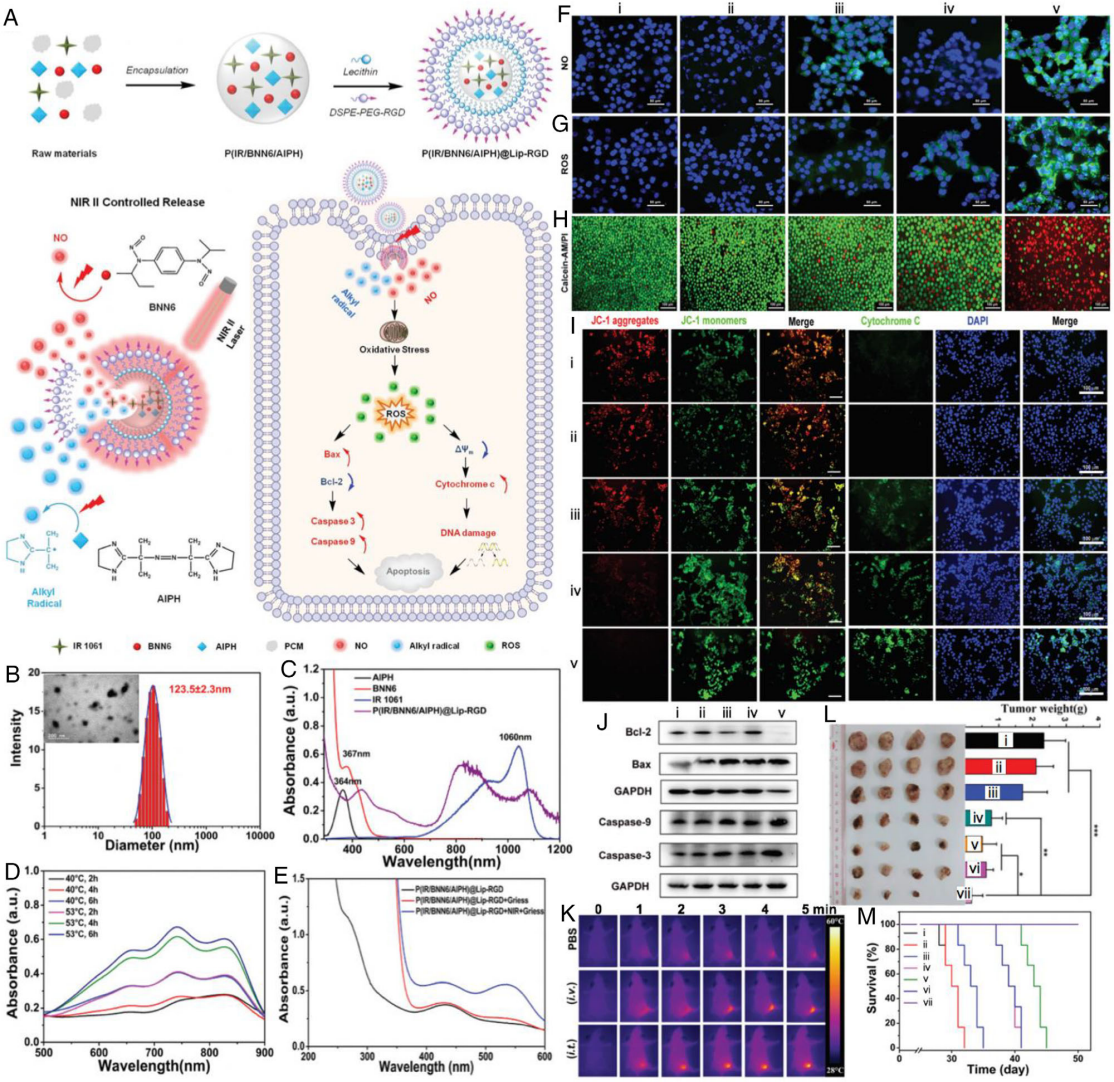
(A) Synthesis of P(IR/BNN6/AIPH)@Lip‐RGD using a simple two‐step method; NIR II‐induced controlled alkyl radical and NO release; synergistic alkyl radical and NO anticancer mechanism of P(IR/BNN6/AIPH)@Lip‐RGD under NIR II laser irradiation. (B) Hydrodynamic diameter of P(IR/BNN6/AIPH)@Lip‐RGD dispersion. Insert: TEM image of P(IR/BNN6/AIPH)@Lip‐RGD. (C) UV–vis spectra of IR1061, BNN6, AIPH and P(IR/BNN6/AIPH)@Lip‐RGD. (D) Detection of alkyl radical release in P(IR/BNN6/AIPH)@Lip‐RGD dispersion under different temperatures and times using ABTS probe. The UV–vis peak in the range of 500–900 nm indicates the presence of alkyl radicals. (E) Detection of NO release in P(IR/BNN6/AIPH)@Lip‐RGD dispersion with and without NIR II laser irradiation using Griess reagent. The UV–vis peak at about 540 nm indicates the presence of NO. (F) Confocal images showing the intracellular NO release level in different treated groups. NO and nucleus were stained by DAF‐FM DA and DAPI emitting green and blue fluorescence, respectively. Scale bars represent 50 µm. (G) Confocal images showing intracellular ROS generation level. ROS and nucleus were stained by DCFH‐DA and DAPI emitting green and blue fluorescence, respectively. Scale bars represent 50 µm. (H) Confocal images showing the live/dead cells percentage after different treatments using a calcein AM and PI dual‐staining method, where live/dead cells were stained by calcein AM/PI displaying green/red color, respectively. Scale bars represent 100 µm. (I) Confocal images showing the damaging effect of different treatments on the mitochondrial membrane, by measuring changes in mitochondrial membrane potential with the use of a JC‐1 assay kit. JC‐1 presents aggregates (red, 525/590 nm) and monomer (green, 485/530 nm) in normal and damaged mitochondria, respectively. Scale bars represent 100 µm; and detection of cytochrome c release of 4T1 cells that underwent different treatments using an immunofluorescence staining method. Cytochrome c and nucleus displayed green and blue fluorescence, respectively. Scale bars represent 100 µm. (J) Western blot data showing the expression level of Bcl‐2, Bax, Caspase 3 and Caspase 9 in 4T1 cells that underwent different treatments. It is noteworthy that (i–v) represent groups with different treatments. (i) PBS, (ii) P(IR)@Lip‐RGD + Laser, (iii) P(IR/BNN6)@Lip‐RGD + Laser, (iv) P(IR/AIPH)@Lip‐RGD + Laser, (v) P(IR/BNN6/AIPH)@Lip‐RGD + Laser. (K) NIR II‐induced in vivo photothermal effect of P(IR/BNN6/AIPH)@Lip‐RGD by i.v. and i.t. injections evaluated on 4T1 tumor‐bearing nude mice. The photothermal effect of mice tumor with an i.v. injection of PBS was used as a control. (L) Tumor size and weight in different groups after 21 days of treatment. (M) Survival rate of nude mice that underwent different treatments. Note that (i–vii) represent groups with different treatments: (i) PBS (i.v.), (ii) P(IR/BNN6/AIPH)@Lip‐RGD (i.t.), (iii) P(IR)@Lip‐RGD (i.t.) + NIR, (iv) P(IR/BNN6)@Lip‐RGD (i.t.) + Laser, (v) P(IR/AIPH)@Lip‐RGD (i.t.) + Laser, (vi) P(IR/BNN6/AIPH)@Lip‐RGD (i.v.) + Laser, and (vii) P(IR/BNN6/AIPH)@Lip‐RGD (i.t.) + Laser. Reproduced with permission.^[^ ^89^ ^]^ Copyright 2021, The Royal Society of Chemistry.

Wu et al. fabricated a biocompatible NIR‐II laser‐driven nanogenerator by encapsulating IR 1061 dye, BNN6, and AIPH into a natural lecithin‐stabilized phase change material. The nanogenerator was further functionalized with an amphiphile (DSPE‐PEG‐RGD) to obtain the final product P(IR/BNN6/AIPH)@Lip‐RGD, which possessed specific tumor targeting ability for synergistic PTT/GT/ART (Figure 10A).^[^ ^89^ ^]^ As shown in Figure 10B, the obtained nanoplatform exhibited a spherical structure and the hydrodynamic diameter was measured to be about 123.5 nm. The successful encapsulation of IR 1061 endowed it with potent absorption in the NIR‐II region for potential PTT (Figure 10C). When exposed to a 1064 nm laser, the photothermal effect generated by IR 1061 triggered a phase change in the nanogenerator, leading to the release of encapsulated BNN6 and AIPH. Subsequently, BNN6 and AIPH decomposed to form highly active NO and alkyl radicals, which were demonstrated by the typical absorption at 540 nm (incubation with Griess reagents) and range of 500–900 nm (incubation with 2,2′‐azinobis(3‐ethylbenzothiazoline‐6‐sulfonic acid), ABTS), respectively (Figure 10D,E). With P(IR/BNN6/AIPH)@Lip‐RGD + Laser treated, 4T1 cells displayed vivid green fluorescence, which revealed that the generation of NO and alkyl radicals contributed to the elevation of ROS (Figure 10F,G). The synergistic therapeutic outcome caused substantial cell death, as identified by the reduced green fluorescence of Calcein‐AM and the increased red fluorescence of/PI (Figure 10H). Mechanistic studies indicated that the synergistic effect induced cancer cell apoptosis through a mitochondria‐mediated apoptotic pathway, which led to severe mitochondrial dysfunction with the release of cytochrome c, down‐regulation of Bcl‐2 protein expression, and activation of apoptosis‐related proteins such as Caspase‐3 and Caspase‐9 (Figure 10I,J). In vivo experiments were conducted based on 4T1 tumor‐bearing nude mice to compare the treatment efficacy of i.v. and i.t. injections of P(IR/BNN6/AIPH)@Lip‐RGD. As shown in Figure 10K–M, the strengthened photothermal effect in the i.t. injection group evidenced the higher nanoplatform concentration at the tumor site, resulting in the most remarkable inhibiting effect with complete suppression of tumor growth and a 100% survival rate. This study provides evidence supporting the effectiveness and safety of a combined strategy involving photothermal‐induced NO/alkyl radical production for cancer therapy.

### 3.4 PTT/GT/RT

RT is a widely used therapeutic method in clinical settings that induces DNA damage through direct ionization and the generation of ROS when exposed to high‐energy X‐ray or γ‐ray radiation.^[^ ^166^, ^167^, ^168^ ^]^ Unlike PTT, RT is not limited by tissue depth, but the hypoxic TME can still hinder its effectiveness.^[^ ^169^ ^]^ It is worth noting that both PTT and NO have the ability to enhance intratumoral blood flow and improve tumor oxygenation, making these hypoxic regions more susceptible to the effects of radiation. By combining these modalities with RT, improved treatment outcomes can be achieved.^[^ ^170^, ^171^, ^172^ ^]^ Moreover, under X‐ray stimulation, the S─NO bond of RSNOs can be cleaved, leading to the release of NO. This allows for precise tumor destruction while minimizing damage to surrounding healthy tissues. Thus, the integration of photothermal radiation therapeutic agents with RSNOs represents a promising strategy.^[^ ^173^ ^]^

**FIGURE 11:**
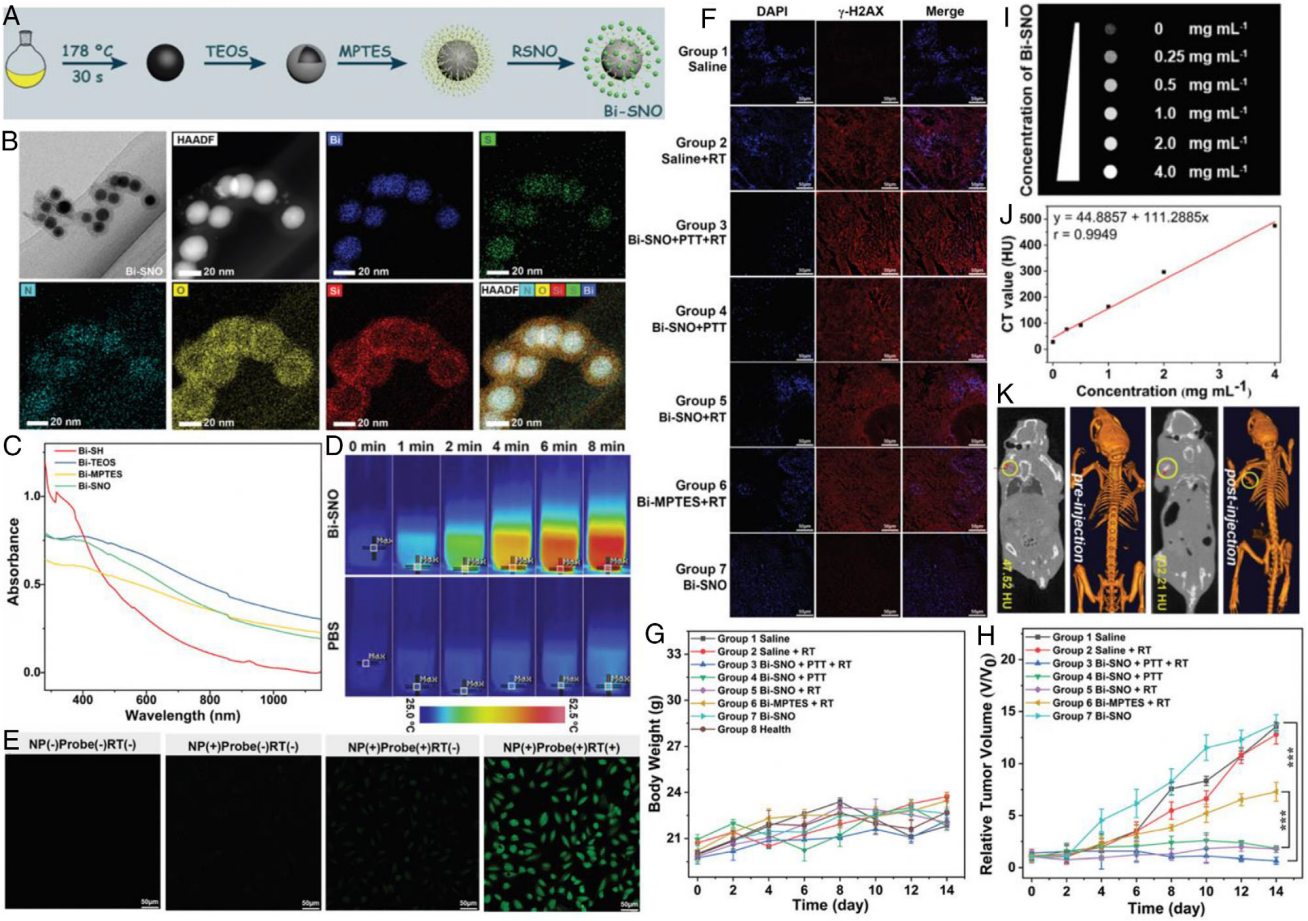
(A) Schematic illustration of the synthetic procedure of Bi‐SNO. (B) TEM images of Bi‐SNO, including elemental mapping images of Bi‐SNO. (C) UV–vis–NIR absorption spectra of Bi at different synthesis stages. (D) Infrared thermal images of Bi‐SNO (300 µg mL^−1^) solution irradiated with an 808 nm laser for 600 s; PBS was set as the control. (E) In the absence of Bi‐SNO, fluorescence probe, and X‐ray radiation; in the presence of Bi‐SNO (300 µg mL^−1^, 1 mL) only; in the presence of Bi‐SNO and fluorescence probe (25 µmol L^−1^, 1 mL) but the absence of X‐ray radiation; in the presence of Bi‐SNO, fluorescence probe, and X‐ray radiation. (The scale bars represent 50 µm). (F) Immunofluorescence staining of the nucleus (DAPI: blue) and the γ‐H2AX (red) for tumor slices in different groups. These images were acquired by CLSM with scale bar 50 µm. (G) Body weights of tumor‐bearing mice in groups 1–7 after therapy and healthy mice in group 8 for 14 days. (H) The relative tumor volume diagram of each group of tumor‐bearing mice after various treatments. (I) In vitro CT images of Bi‐SNO solution with various concentrations. (J) The CT value of Bi‐SNO in accordance with different concentrations. (K) In vivo CT imaging of the tumor pre‐injection and post‐injection. Reproduced with permission.^[^ ^174^ ^]^ Copyright 2020, The Royal Society of Chemistry.

For example, Zhang et al. constructed a RSNO functionalized versatile bismuth (Bi)‐based nanoplatform (denoted as Bi‐SNO) for cooperative PTT/RT/GT (Figure 11A).^[^ ^174^ ^]^ As shown in Figure 11B, the core‐shell structured Bi‐SNO possessed good monodispersity with an average particle size of ≈36 nm, and the corresponding elemental mapping images further confirmed the morphology and compositions. The strong optical absorption in the NIR region indicated the feasibility of Bi‐SNO as a good PTA (PCE = 15.5%) (Figure 11C). After 808 nm laser irradiation for 8 min, the Bi‐SNO aqueous dispersion (300 µg mL^−1^) showed a rapid temperature increment from room temperature to 52.0°C, while the temperature of the PBS group only increased by 1.8°C (Figure 11D). In the presence of X‐ray radiation, Bi‐SNO was able to break down the S─NO bonds and simultaneously trigger a large amount of NO (over 60 µm), giving rise to vivid intracellular green fluorescence after co‐incubation with the DAF‐FM DA (Figure 11E). As shown in Figure 11F, the NO‐sensitized RT (Bi‐SNO + RT) was verified by the more severe damage of double‐stranded DNA with increased nuclei shrinkage and γ‐H2AX expression. Meanwhile, the 808 nm laser treatment further enlarged the curative effects due to the triple‐modal therapies. As for in vivo experiments, no significant fluctuations in body weight among all the tumor‐bearing mice demonstrated the minor side effects of these treatments (Figure 11G). Upon exposure to both X‐ray and 808 nm laser, the Bi‐SNO could achieve synergistic PTT/GT/RT to completely destroy the tumors (Figure 11H). Benefiting from the high X‐ray absorption coefficient, the capability of Bi‐SNO as a potential CT contrast agent was also conducted. The signal intensity displayed a concentration‐dependent manner, and the corresponding CT values (Hounsfield units, HU) were recorded to give a high slope of 111.29  ± 5.67 (Figure 11I,J). After injection of Bi‐SNO, the CT value of the tumor site increased sharply from 47.52 HU to 232.21 HU, revealing the outstanding imaging performance in vivo (Figure 11K).

### 3.5 PTT/GT/IT

IT has emerged as a highly promising field in biomedicine, generating significant research interest due to its ability to harness the immune system and induce robust systemic antitumor immune responses.^[^ ^135^, ^175^, ^176^ ^]^ Within the TME, tumor‐associated macrophages (TAMs) and regulatory T (Treg) cells play pivotal roles in tumor growth and metastasis. TAMs, as immune cells infiltrating the TME, are among the most abundant non‐neoplastic immune cell types in various tumors. Typically, TAMs exhibit an M2 phenotype, secreting anti‐inflammatory cytokines and displaying immunosuppressive and tumor‐promoting functions.^[^ ^177^ ^]^ In contrast, M1 macrophages possess pro‐inflammatory properties and are associated with an enhanced immune response. M1 macrophages secrete pro‐inflammatory cytokines and activate cytotoxic CD8^+^ T cells, thereby promoting an antitumor immune response. Consequently, therapeutic strategies that aim to modulate the TME toward a pro‐inflammatory environment, promote CD8^+^ T cell infiltration, and limit immunosuppressive cell populations (such as M2 macrophages and Tregs) are actively being explored in cancer IT.^[^ ^178^, ^179^ ^]^ As for nanomedicines, two specific areas have garnered attention in recent years, that is, ICD and immune checkpoint blockade (ICB).^[^ ^180^, ^181^ ^]^ ICD refers to a form of cell death that triggers an immune response by releasing danger signals and activating the immune system. Various cancer treatments, including PTT, PDT, chemotehrapy, and RT, have been reported to induce ICD. During ICD, dying cancer cells release molecules such as damage‐associated molecular patterns (DAMPs) and tumor‐associated antigens (TAAs), activating the immune system to eliminate cancer cells and generate an antitumor immune memory.^[^ ^182^, ^183^, ^184^ ^]^

**FIGURE 12:**
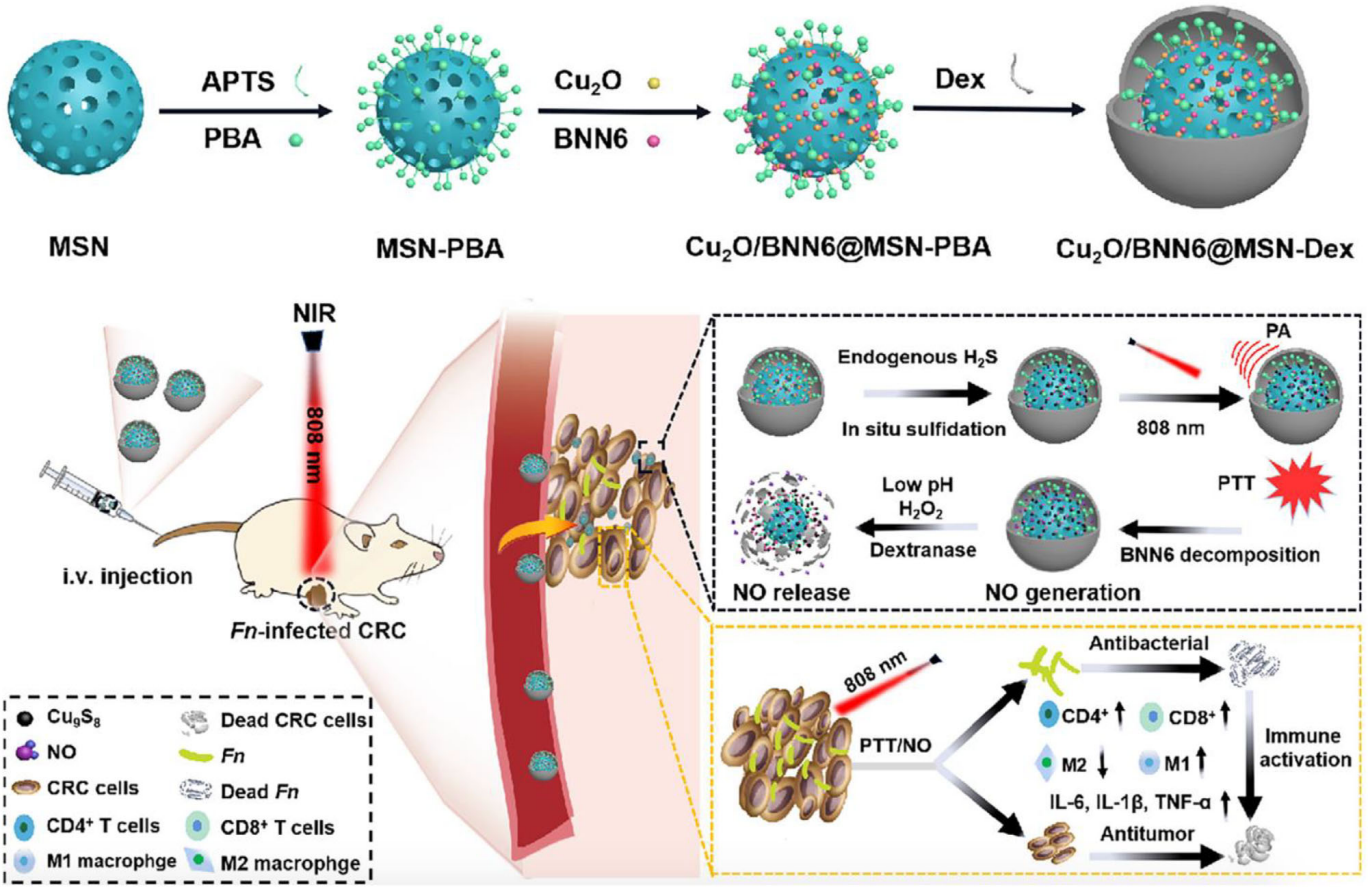
Preparation and structure of Cu_2_O/BNN6@MSN‐Dex and treatment procedure and mechanism of in situ‐activated Cu_2_O/BNN6@MSN‐Dex against Fn infected CRC by synchronous intratumor Fn elimination and anti‐tumor immune activation under PA imaging‐guided photothermal and NO gas combinatorial therapy. Reproduced with permission.^[^ ^188^ ^]^ Copyright 2023, Elsevier Inc.

Nowadays, PTT and NO gas, in addition to their local effects on the treated tumor, have also been demonstrated to induce systemic immune responses. This systemic immune activation can lead to the control of distant metastases and provide long‐term protection against tumor recurrence.^[^ ^185^ ^]^ Fusobacterium nucleatum (Fn) is a bacterium commonly found in the human gut microbiota, promoting tumor growth and progression, particularly in colorectal cancer (CRC). The presence of Fn in TME has been linked to various mechanisms that contribute to drug resistance.^[^ ^186^, ^187^ ^]^ In a recent study, Xin et al. proposed an in situ‐activated nanoplatform, Cu_2_O/BNN6@MSN‐Dex, for selective tumor inhibition and synchronous elimination of the CRC‐resident pathogenic bacterium Fn through combined PTT and GT (Figure 12).^[^ ^188^ ^]^ This nanoplatform was constructed by loading cuprous oxide (Cu_2_O) and BNN6 into dextran (Dex)‐decorated MSN, in which the formed boronate linkage between phenylboronic acid (PBA) and Dex enabled pH‐/H_2_O_2_‐sensitive performance. Once it reached the tumor site, the overexpressed endogenous H_2_S sulfuretted the loaded Cu_2_O, transforming it into Cu_9_S_8_. Under 808 nm laser irradiation, the nanoplatform generated local hyperthermia, enabling PA imaging, non‐invasive PTT, as well as NO generation. The Dex coating on the nanoplatform was cleaved via pH‐ and H_2_O_2_‐mediated mechanisms, allowing the release of NO to reinforce PTT and ablate both the tumor cells and Fn. Moreover, the PTT/NO treatment could reverse the Fn‐mediated immunosuppressive microenvironment and successfully provoke the activation of antitumor immunity, which was reflected in the significant increase of M1 macrophages and CD4^+^ and CD8^+^ T cells. This study not only demonstrates an effective approach for PA imaging‐guided PTT/GT with in situ activation mechanisms but also provides a valid strategy for enhanced CRC treatment by specifically eliminating intratumor pathogens and stimulating antitumor immune responses.

ICB is a specific type of IT using drugs called immune checkpoint inhibitors to block inhibitory molecules on immune cells, known as immune checkpoints (e.g., programmed cell death protein 1 (PD‐1) and cytotoxic T‐lymphocyte‐associated protein 4 (CTLA‐4), etc.).^[^ ^189^ ^]^ Immune checkpoints are able to regulate immune responses and prevent excessive immune activation, but cancer cells can hijack these checkpoints to evade immune detection and attack. ICB drugs, such as anti‐PD‐1 or anti‐CTLA‐4 antibodies, disrupt the interaction between immune checkpoints and their ligands, allowing the immune system to mount a stronger antitumor response.^[^ ^190^, ^191^ ^]^ NO gas can help modulate the TME by reducing immunosuppression and promoting an immune‐supportive milieu. Except for PTT/NO‐induced ICD, ICB can further enhance the presentation and recognition of these tumor antigens, increasing the activation of tumor‐specific immune responses. Therefore, the combination of PTT, NO gas with immune checkpoint inhibitors is expected to offer a powerful approach to maximize the immune response, overcome tumor resistance, and improve treatment outcomes in a synergistic manner.^[^ ^47^, ^192^ ^]^

**FIGURE 13:**
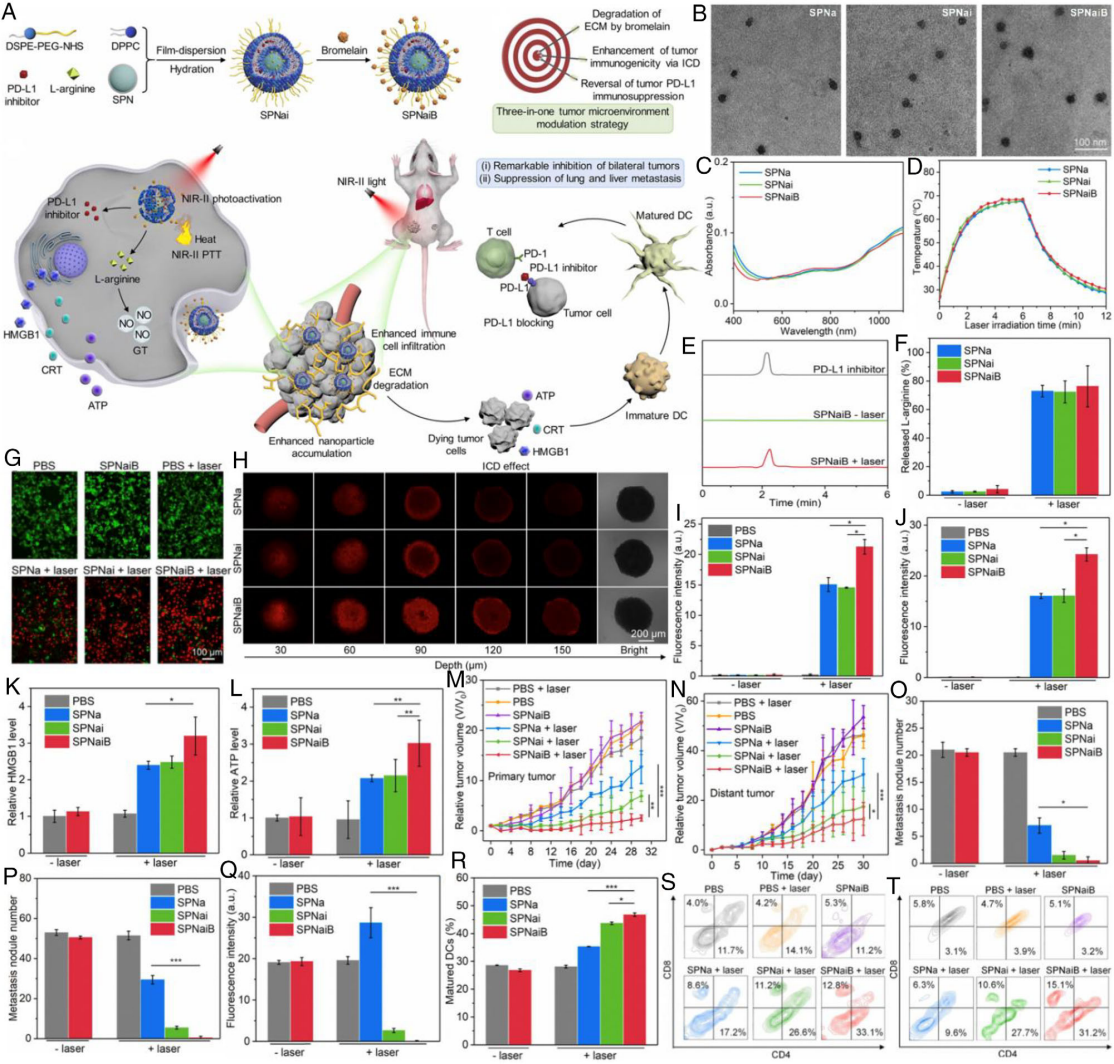
(A) Schematic illustration of the formation of SPNaiB via film‐dispersion, hydration, and modification; summary of the modulation of immunosuppressive TME via a three‐in‐one strategy; schematic illustration of the mechanism of SPNaiB for combinational IT. (B) TEM images of SPNa, SPNai, and SPNaiB. (C) UV–vis–NIR spectra of SPNa, SPNai, and SPNaiB. (D) Temperature curves of solutions containing SPNa, SPNai, and SPNaiB under exposure to NIR‐II laser (1064 nm, 1.0 W cm^−2^) for different times. (E) The release analysis of PD‐L1 inhibitors from SPNaiB without and with exposure to laser for 5 min. (F) The release percentages of L‐Arg from nanomediators before and after 1064 nm laser irradiation for 5 min. (G) Dead and living fluorescence staining images of 4T1 cells. (H) Fluorescence images of 3D multicellular tumor spheroids treated with fluorescence‐labeled SPNa, SPNai, and SPNaiB at different depths. (I) Mean fluorescence intensity of NO staining for 4T1 cells after different treatments. (J) CRT staining fluorescence intensity for 4T1 cells. (K) Extracellular HMGB1 levels measured by ELISA kit after treatments of 4T1 cells with PBS, SPNa, SPNai, or SPNaiB with or without exposure under laser. (L) Extracellular ATP levels measured by ELISA kit after treatments of 4T1 cells with PBS, SPNa, SPNai, or SPNaiB with or without exposure under laser. (M) The relative volume of primary tumors in various treated mice at appointed days. (N) The relative volume of distant tumors in various treated mice at appointed days. (O) Tumor metastasis analysis in lungs for various treated mice. (P) Tumor metastasis analysis in a same area of livers for various treated mice. (Q) PD‐L1 staining fluorescence intensity of tumors in various treated mice. (R) Level of matured DCs in various treated groups. (S) Evaluation of CD4^+^ and CD8^+^ T cells in primary tumors from various treated mice by flow cytometry. (T) Evaluation of CD4^+^ and CD8^+^ T cells in distant tumors from various treated mice by flow cytometry. Reproduced with permission.^[^ ^193^ ^]^ Copyright 2023, Elsevier Inc.

Li et al. developed novel nanomediators (denoted as SPNaiB) that contained an inside core with encapsulations of NIR‐II absorbing SPNs, PD‐L1, and L‐Arg, and a thermal‐responsive shell with modification of bromelain to modulate the TME for combined IT (Figure 13A).^[^ ^193^ ^]^ For comparison, SPNai (without the bromelain) and SPNa (without the bromelain and L‐Arg) were also synthesized. As shown in Figure 13B, TEM images indicated that all nanoparticles were spherical with a uniform distribution. The SPNa, SPNai, and SPNaiB similarly exhibited a strong absorbance in the NIR‐II region, and the remarkable temperature elevation verified their excellent photothermal conversion properties under 1064 nm laser irradiation (Figure 13C,D). Moreover, the generated hyperthermia could trigger the collapse of the thermal‐responsive component, leading to the release of PD‐L1 inhibitors and L‐Arg (Figure 13E,F). The percentages of dead cells in the SPNa + laser, SPNai + laser, and SPNaiB + laser groups were obviously increased, suggesting enhanced anticancer efficacy (Figure 13G). Through ICG labeling, SPNaiB was found to display a stronger penetrating ability than their control counterparts, which could be attributed to the bromelain‐induced specific degradation of collagen in the tumor's extracellular matrix (ECM) (Figure 13H). Accordingly, SPNaiB + Laser group exhibited the strongest green fluorescence, indicating the abundant production of NO gas (Figure 13I). Both NIR‐II PTT and GT could trigger ICD in dying cancer cells, which was reflected in the upregulated levels of ATP, calreticulin (CRT), and high mobility group box 1 protein (HMGB1) (Figure 13J–L). In vivo therapeutic experiments showed that the SPNaiB + Laser group achieved 8.4‐fold and 3.7‐fold inhibition of primary and distant tumors, respectively (Figure 13M,N), as well as excellent antimetastasis efficacy due to the infiltration of immune cells into the lungs and livers (Figure 13O,P). More importantly, NIR‐II light‐activation of SPNai and SPNaiB could release PD‐L1 inhibitors to effectively block the PD‐L1 immunosuppressive pathway, boosting the matured dendritic cells (DCs) (Figure 13Q,R). As a consequence, the levels of CD4^+^ (33.1% and 31.2%) and CD8^+^ (12.8% and 15.1%) T cell in primary and distant tumors showed the biggest increases after SPNaiB + Laser treatment (Figure 13S,T). This comprehensive approach synergistically amplifies the antitumor immune response by degrading the ECM to promote nanoparticle accumulation and immune cell infiltration, triggering ICD through NIR‐II PTT and GT to enhance tumor immunogenicity, and blocking PD‐L1 immunosuppression using PD‐L1 inhibitors, resulting in a significant inhibition of tumor growth and suppression of liver and lung metastasis in mouse models with subcutaneous bilateral 4T1 tumors.

## 4 PTT/GT‐BASED TETRA‐MODAL THERAPY

Apart from triple‐modal therapy, researchers have explored the concept of tetra‐modal therapy, which combines four different therapeutic modalities, to offer a more comprehensive and potent approach to cancer treatment. By incorporating multiple modalities, tetra‐modal therapy can target diverse cellular pathways and molecular targets, providing comprehensive coverage and addressing the heterogeneity of cancer cells within a tumor. This approach also has the potential to allow for lower individual doses of each treatment component, thereby minimizing systemic toxicity and adverse effects on healthy tissues. The synergy between the different modalities can enhance the overall therapeutic outcome and potentially improve long‐term cancer control and patient survival rates. Tetra‐modal therapy represents an exciting avenue for advancing cancer treatment strategies and improving patient outcomes.^[^ ^194^, ^195^, ^196^ ^]^ In this section, several tetra‐modal treatments such as PTT/GT/CHT/PDT, PTT/GT/CHT/CDT, PTT/GT/PDT/IT, PTT/GT/ST/IT, PTT/GT/Ca^2+^ overload/IT, PTT/GT/FT/IT, and PTT/GT/CDT/IT are introduced.

### 4.1 PTT/GT/PDT/CHT

PTT, NO‐mediated GT, PDT, and CHT can all be performed at specific tumor sites. By combining these modalities, multiple pathways can be targeted simultaneously, resulting in a synergistic effect and improved treatment outcomes by overcoming resistance mechanisms employed by tumor cells and tumor heterogeneity. Besides, such integration is capable of enhancing the sensitivity of cancer cells to CHT, allowing for effective tumor control with lower drug concentrations, thus the potential for side effects and toxicity can be minimized.

As cyanine dyes are capable of inducing both PDT and PTT under single laser irradiation, the combination of such photosensitive agents with NO donors and chemotherapeutic agents has been very popular to realize multi‐modal cancer treatment in recent years. For example, Liu et al. developed advanced functional polymersomes with a high amount of NO donors (nitrate, ─ONO_2_) (3.94 µmol NO/mg of polymersomes) grown on the polymer chains, which further simultaneously carried a hydrophobic heptamethine dye, IR780, on the membrane layer and a hydrophilic chemotherapeutic agent, DOX⋅HCl, in the lumen (denoted as NPS_D‐IR_).^[^ ^197^ ^]^ The release of NO and DOX from the NPS_D‐IR_ was triggered by TME, and accelerated by remote NIR light irradiation due to the photothermal effect of IR780. The high‐concentration of NO inhibited the expression of P‐gp, sensitizing the tumor cells to CHT by overcoming MDR. Meanwhile, the IR780 could also generate abundant ROS to induce PDT under 808 nm laser irradiation. This combination approach showed superior treatment efficacy, leading to the complete eradication of tumors with minimal side effects.

Jing et al. fabricated a nanovesicle system called PIDA, which integrated PEGylated ICG (mPEG‐ICG), DOX⋅HCl, and L‐Arg in the presence of PEP, enabling multimodal therapy to sensitize drug‐resistant cancers.^[^ ^198^ ^]^ When exposed to 808 nm irradiation, PIDA nanovesicles not only generated mild heat for PTT (PCE = 13.8%), but also promoted the production of NO due to the reaction of ROS and L‐Arg. The released NO caused sensitization of K562/ADR cells to DOX⋅HCl via mitochondrial dysfunction, which was reflected in the decrease in mitochondrial membrane potential, increase in ROS, and significant depletion of ATP. These combined effects brought significant anticancer efficacy, a tumor inhibition rate of 80.8% was obtained based on K562/ADR‐bearing nude mice. This research provides valuable insights for the design of nanoplatforms aimed at treating drug‐resistant cancers.

### 4.2 PTT/GT/CHT/CDT

CDT is an emerging therapeutic strategy for cancer treatment that utilizes chemical reactions to induce cytotoxic effects. Unlike traditional therapies, including CHT, RT, or PDT, which rely on the direct interaction of drugs, radiation, or light with cells, CDT employs the generation of toxic ROS through chemical reactions. It typically involves the utilization of transition metal ions, such as Fe^2+^, Cu^+^_,_ or Mn^2+^ to catalyze H_2_O_2_ to produce highly reactive hydroxyl radicals (•OH) via Fenton or Fenton‐like reactions.^[^ ^199^, ^200^ ^]^ Tumors often exhibit elevated levels of H_2_O_2_ due to increased metabolic activity and dysfunctional redox regulation. By introducing catalytic metal ions into the tumor, CDT takes advantage of the overproduced H_2_O_2_ to selectively generate ROS, targeting cancer cells while sparing healthy tissues. PTT and NO gas are able to enhance the efficacy of CDT and CHT by increasing Fenton agents and chemotherapeutic drug concentrations at the tumor site. The combination of PTT, GT, CDT, and CHT can help overcome resistance mechanisms by targeting cancer cells through multiple pathways. Moreover, these four treatment modalities can all induce oxidative stress in cancer cells and modulate TME in a synergistic manner, leading to enhanced tumor cell death and improved therapeutic outcomes.^[^ ^201^, ^202^ ^]^

In recent years, manganese dioxide (MnO_2_) nanomaterials have gained significant attention in CDT because they can deplete intracellular GSH, an important antioxidant molecule that helps protect cells from oxidative stress.^[^ ^203^, ^204^ ^]^ The design of MnO_2_ nanomaterials with different morphologies allows for tailoring their reactivity, surface area, drug loading capacity, and biocompatibility, enabling their use in a wide range of biomedical applications. Zhang et al. constructed a multifunctional nanoplatform (denoted as MAPRF) based on PDA‐coated hollow mesoporous manganese dioxide (H‐MnO_2_), which was loaded with the histone deacetylase inhibitor N‐(2‐amino‐5‐fluorophenyl)−4‐(1H‐imidazo [4,5‐f][1,10]phenanthrolin‐2‐yl)benzamide (AFIPB) inside the H‐MnO_2_ and ruthenium nitrosyl donor (Ru‐NO)/folic acid (FA) outside the PDA shell.^[^ ^205^ ^]^ The MAPRF was found to: (1) preferentially accumulate in tumor cells due to the FA targeting group; (2) demonstrate a good photothermal effect and controlled release of NO under 808 nm laser irradiation for PTT/GT; and (3) deplete GSH to trigger the release of AFIPB at the tumor site for CHT and disrupt the antioxidant defense system. As a result, the released Mn^2+^ ions from H‐MnO_2_ enabled MR imaging and CDT, further enhancing the therapeutic effects. In summary, the multifunctional nature of the nanoplatform, including targeted delivery, controlled NO/drug release, photothermal effect, TME responsiveness, and additional MR imaging and CDT capabilities, makes it a valuable approach for enhancing the efficacy of anticancer treatment.

### 4.3 PTT/GT/PDT/IT

The advantages of the synergy between PTT/NO‐induced GT and PDT have been presented earlier in the article. It is noticeable that both PTT, NO gas, and PDT can induce ICD and modulate the TME to promote an immune‐supportive milieu, thus boosting anti‐tumor immune responses.^[^ ^206^, ^207^, ^208^ ^]^ Ding et al. described a nanoplatform (denoted as CMH‐OBN) composed of Ce6‐melanin‐hyaluronic acid (Ce6‐MNP‐HA, CMH) nanoparticles and benzofuroxan‐encapsulated oxidized bletilla striata polysaccharide microcapsules (OBN) for combined PTT/GT/PDT/IT.^[^ ^148^ ^]^ Due to the presence of melanine and Ce6, the CMH component could generate hyperthermia and a large number of ROS for PTT and PDT. The OBN microcapsules could release NO gas through cascade reactions in the presence of ROS, laser irradiation, and high expression of GSH. NO was able to up‐regulate the soluble guanylate cyclase‐cyclic guanosine monophosphate (sGC‐cGMP) signaling pathway, which helped relieve hypoxia for enhanced PDT. Furthermore, the cascaded release of ROS and NO led to the formation of ONOO^−^ with higher lethality. These combined therapies also initiated cellular apoptotic procedures and activated T cells at the tumor sites to promote IT. Additionally, the CMH‐OBN nanoplatform offered MR imaging and infrared photothermal imaging guidance, enabling visualization and targeted treatment.

**FIGURE 14:**
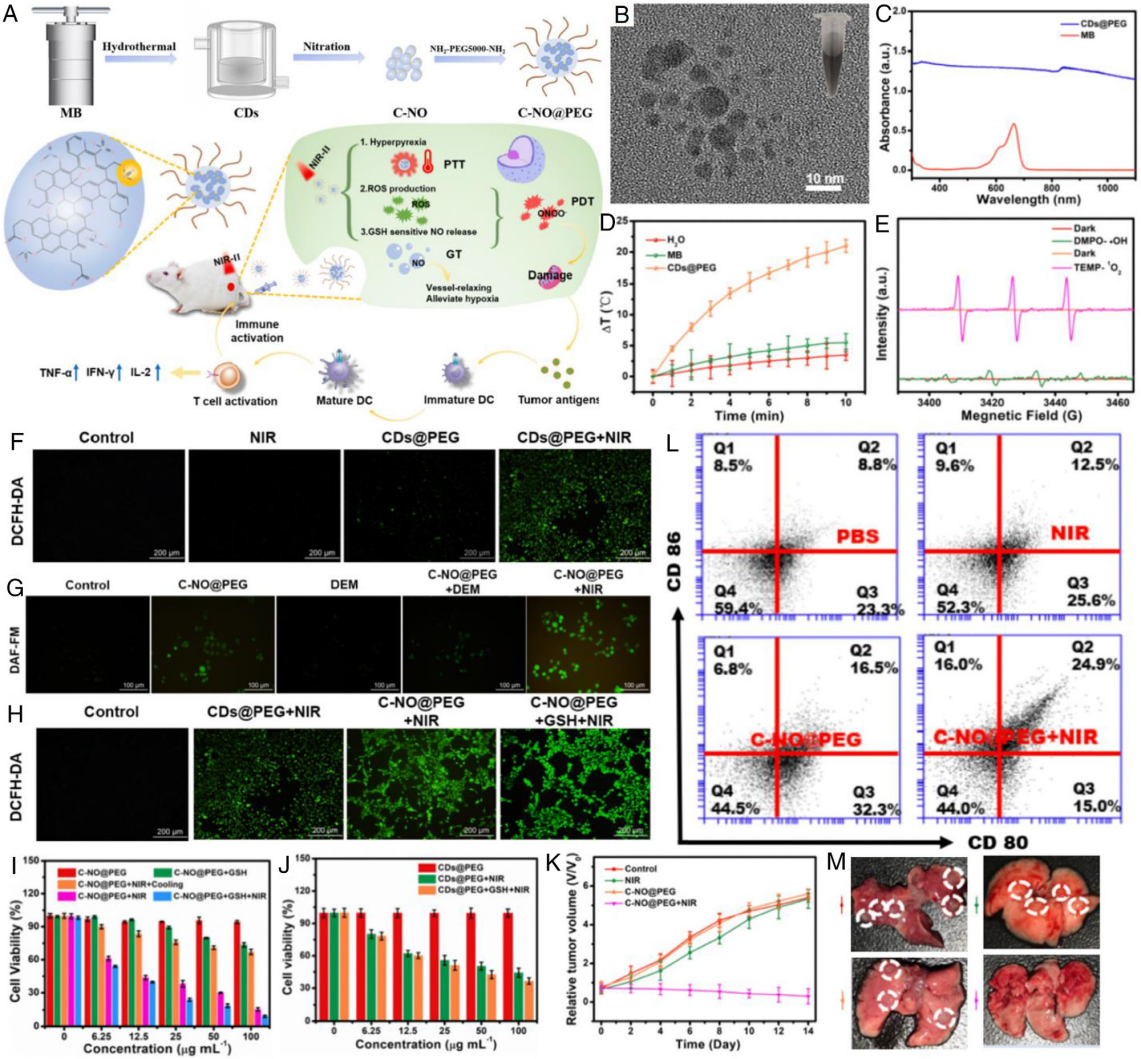
(A) The illustration of converting process from MB to CDs. (B) TEM image (inset image: CDs aqueous dispersion). (C) The UV–vis spectra of the aqueous solutions of CDs@PEG and MB. (D) Photothermal heating curves of different samples with different concentrations under 1064 nm laser irradiation. (E) ESR spectra of DMPO and TEMP treated by CDs with/without irradiation. (F) Fluorescence images of 4T1 cells and DCFH‐DA treated by CDs@PEG with/without irradiation. (G) The fluorescence imaging of 4T1 cells treated by C‐NO@PEG by using DAF‐FM as NO probe. (H) Fluorescence images of DCFH‐DA stained 4T1 cells under different conditions. MTT assay of 4T1 cells treated by (I) C‐NO@PEG and (J) CDs@PEG under different conditions. (K) Tumor volumes of mice of all groups during the treatments. (L) Quantification of CD80 and CD86 expression after different treatments by flow cytometry. (M) The photographs of lung nodules in different groups after the treatments. Reproduced with permission.^[^ ^209^ ^]^ Copyright 2022, Elsevier Inc.

Wang et al. reported simple pegylated nitrated carbon dots (C‐NO@PEG) that integrated GT and phototherapy into a single nanoparticle (Figure 14A).^[^ ^209^ ^]^ The carbon nanodots (CDs) were initially synthesized using methylene blue (MB) as a carbon source through a hydrothermal strategy, with sulfuric acid (H_2_SO_4_) serving as an oxidizing and dehydrating agent for carbonization. On account of the abundant ─OH of CDs, the nitration reaction was carried out to synthesize ─ONO_2_ modified carbon nanodots (C‐NO) as NO donor, followed by NH_2_‐PEG_5000_‐NH_2_ modification (C‐NO@PEG). As shown in Figure 14B,C, the obtained CDs with an average particle size of 5–10 nm possessed excellent NIR‐II light absorption, making them suitable for phototherapy. Upon 1064 nm laser (0.8 W cm^−2^) irradiation for 10 min, the CDs@PEG exhibited a fast temperature increase (Δ*T* = 21°C) compared to the MB and pure water groups, and a PCE of 29.7% was also calculated (Figure 14D). Attributed to the presence of O_2_ and photo‐excited electrons, the CDs@PEG could generate massive ROS, including ^1^O_2_ and •OH, when exposed to a laser (Figure 14E). Consistently, the vivid green fluorescence in the 4T1 cells was noticed after CDs@PEG plus laser treatment, indicating the PDT efficacy of CDs@PEG (Figure 14F). In view of Figure 14G, NO was released from C‐NO@PEG accompanied by GSH consumption, which was evidenced by the disappearance of fluorescence after diethyl maleate (DEM, GSH shielding agent) addition. Moreover, the photothermal effect was also able to boost the NO release, which in situ reacted with ROS to form highly cytotoxic ONOO^−^ (Figure 14H). Taken together, the combined PTT/PDT with NO gas resulted in about 90.4% of 4T1 cell death and complete eradication of 4T1 tumors in vivo (Figure 14I–K). Additionally, the treatment induced an immune response with an enhanced DC maturation percentage to efficiently inhibit tumor metastasis (Figure 14L,M).

### 4.4 PTT/GT/ST/IT

ST is a therapeutic approach aimed at depriving tumor cells of essential nutrients required for their growth and survival. It exploits the metabolic vulnerabilities of cancer cells by targeting specific metabolic pathways or altering the TME to restrict nutrient availability.^[^ ^210^, ^211^ ^]^ Up to now, there are several strategies employed in ST, such as anti‐angiogenesis, metabolic inhibitors, and nutrient restriction. Among them, blocking the supply of glucose by specific metabolic enzymes (e.g., glucose oxidase, GOx) to limit the energy required for tumor cell proliferation has become a promising strategy, as the generated products, H_2_O_2_ and gluconic acid, can also be used in combination with other treatment modalities.^[^ ^212^, ^213^ ^]^ The photothermal effect and hypoxia relief resulting from PTT and NO gas are beneficial to the decomposition of glucose for ST, as the GOx‐based glycolysis process is O_2_‐dependent. By uniting these modalities, metabolic stress in the tumor can be synergistically increased, leading to enhanced tumor cell death and improved treatment outcomes. Similarly, the combination of PTT, GT, and ST is capable of overcoming immune suppression and resistance, thus achieving improved immune recognition and response.^[^ ^214^, ^215^ ^]^

For example, Huang et al. proposed a novel nanoplatform (M@BPAG) that combined black phosphorus nanosheets (BP), L‐Arg, and GOx with macrophage cell membranes coated for highly efficient glioblastoma (GBM) treatment.^[^ ^216^ ^]^ BP was chosen for the excellent biocompatibility, biodegradability, low toxicity, high PCE, and surface‐to‐volume ratio, but it lacked stability when exposed to air and moisture. In this design, L‐Arg was first introduced to the surface of BP (BPA) through an esterification reaction, which not only served as a source of NO but also prevented further oxidative degradation of BP. To stimulate the release of NO, GOx was then linked to L‐Arg through amidation, resulting in the formation of BPAG. Last, macrophage membranes were coated on the BPAG (M@BPAG) to facilitate penetration through the blood‐brain barrier (BBB) and improve GBM‐targeting ability. After oxidizing glucose using the catalysis of GOx, massive H_2_O_2_ could be formed to further promote the generation of NO through the oxidation of L‐Arg. Besides, the release of H_2_O_2_ and NO could be significantly accelerated by 808 nm NIR irradiation. Remarkably, the M@BPAG + Laser group was found to convert cold tumors to hot ones by boosting the CD8^+^ T cells and M1 macrophage infiltration while reducing the infiltration M2 macrophages and PD‐L1 expression. As expected, based on the orthotopic GBM mouse model, the M@BPAG + Laser group exhibited the strongest antitumor activity without causing systemic toxicity compared to other treatment groups. This work offers an effective approach to reprogram the TME via multi‐modal targeted therapy, broadening the potential clinical application of BP nanosheets.

### 4.5 PTT/GT/Ca^2+^ overload/IT

Intracellular Ca^2+^ ions, primarily stored in mitochondria and the endoplasmic reticulum, play a crucial role in various cellular processes. However, excessive Ca^2+^ accumulation in cells can lead to oxidative stress and subsequent cell apoptosis or necrosis.^[^ ^217^, ^218^ ^]^ As such, Ca^2+^ overload has been explored as a strategy to selectively induce cytotoxicity in cancer cells. Calcium peroxide (CaO_2_) is known for its environmentally friendly properties and has been widely used in disinfection and contaminant degradation applications. Due to its pH sensitivity and ability to supply intracellular Ca^2+^ ions, H_2_O_2_, and O_2_, recent studies have indeed explored the potential of nanostructured CaO_2_ in various biomedical applications.^[^ ^219^, ^220^ ^]^ By taking advantage of these properties, CaO_2_ can: (1) serve as a smart drug carrier; (2) alleviate tumor hypoxia to overcome MDR or enhance the PDT efficacy; (3) promote CDT or NO generation when combining with Fenton/Fenton‐like nanoagents or L‐Arg; (4) induce cancer cells death via Ca^2+^ overload. Considering its poor stability in aqueous solutions, the surface modification and functionalization of CaO_2_ are urgently needed.

**FIGURE 15:**
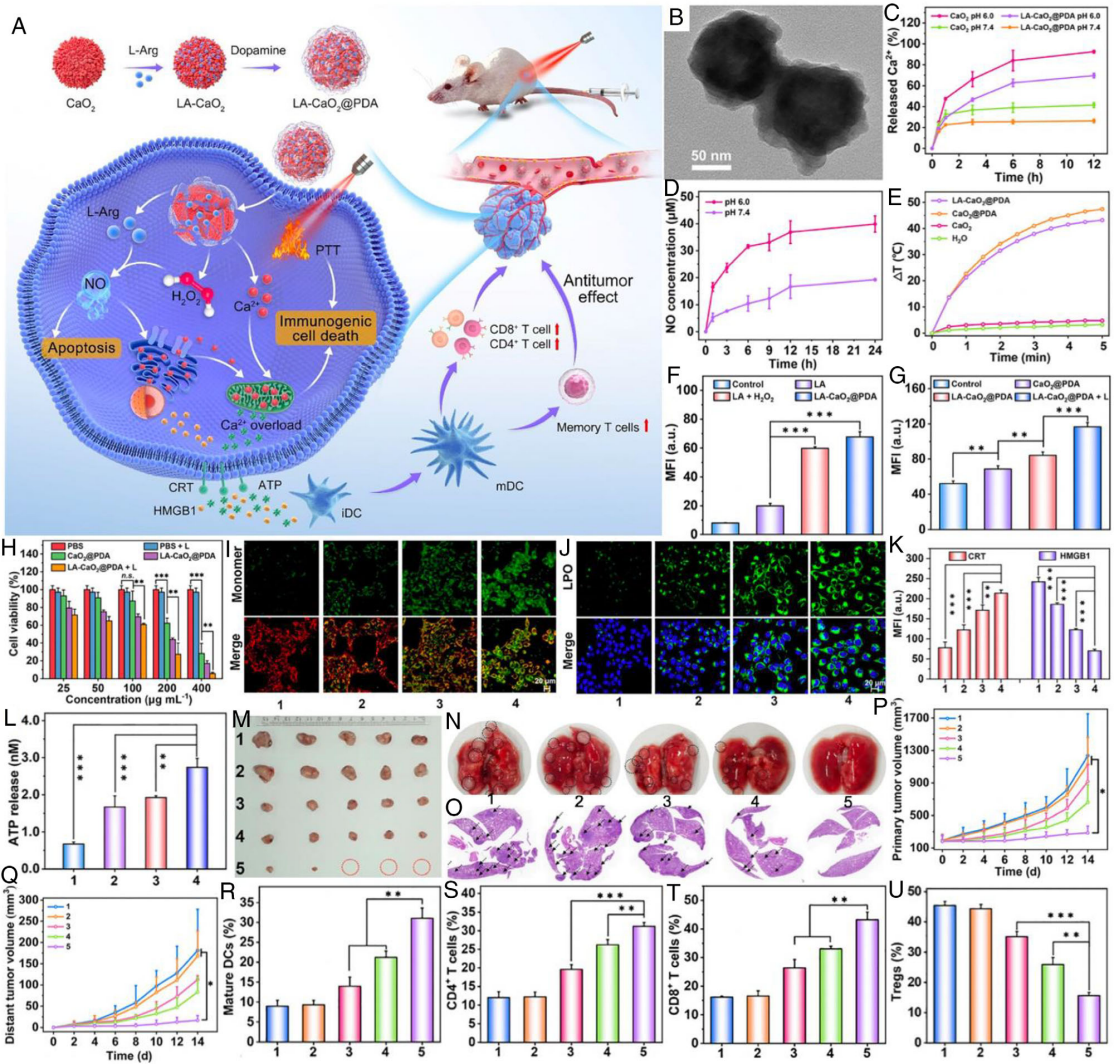
(A) Schematic illustration of the synthesis and antitumor therapeutic mechanism of LA‐CaO_2_@PDA. (B) TEM image of LA‐CaO_2_@PDA. (C) Ca^2+^ release profile from CaO_2_ and LA‐CaO_2_@PDA under different pH values (7.4 or 6.0). (D) pH responsive NO release from LA‐CaO_2_@PDA under different pH values (7.4 or 6.0). (E) Photothermal heating curves of pure water, CaO_2_, CaO_2_@PDA, and LA‐CaO_2_@PDA under 808 nm laser irradiation (1 W cm^−2^, 5 min). (F) Quantitative analysis of intracellular NO level stained with DAF‐FM DA. (G) Quantitative analysis of intracellular Ca^2+^ concentration via Fluo 4‐AM staining. (H) Cell viability of 4T1 cells after various treatments determined by CCK‐8 assay. **p < 0.01, ***p < 0.001. n.s., indicates no significance. (I) The change of mitochondrial membrane potential and (J) the peroxidation of membrane lipids in 4T1 cells after different treatments assessed by JC‐1 staining and LPO staining, respectively. (K) Quantitative analysis of CRT exposure and HMGB1 release. (L) Detection of ATP extracellular secretion. **p < 0.01, ***p < 0.001. Groups (1–4) represents Control, CaO_2_@PDA, LA‐CaO_2_@PDA, and LA‐CaO_2_@PDA + Laser, respectively. (M) Photographs of tumors in each group at the end of treatments. (N) Representative photographs and (O) H&E staining of lung tissues of 4T1 tumor‐bearing mice at the end of treatments. (P) Primary and (Q) distant tumor growth curves of 4T1 tumor bearing mice after different treatments. Quantitative analysis of DC maturation (R) (CD11C^+^CD80^+^CD86^+^), (S) tumor infiltrating CD4^+^ T lymphocytes (CD3^+^CD4^+^), (T) tumor infiltrating CD8^+^ T lymphocytes (CD3^+^CD8^+^), and (U) tumor infiltrating Tregs (CD4^+^Foxp3^+^) in tumor‐draining lymph nodes after various treatments. *p < 0.05, **p < 0.01, ***p < 0.001. Groups (1–5) represents PBS, PBS + Laser, CaO_2_@PDA, LA‐CaO_2_@PDA, and LA‐CaO_2_@PDA + Laser, respectively. Reproduced with permission.^[^ ^221^ ^]^ Copyright 2022, Elsevier Inc.

For example, Hao et al. prepared PDA‐coated L‐Arg‐loaded mesoporous CaO_2_ nanoparticles (LA‐CaO_2_@PDA) for Ca^2+^ overload/NO‐enhanced photothermal‐immuno treatment of breast cancer (Figure 15A).^[^ ^221^ ^]^ As shown in Figure 15B, the LA‐CaO_2_@PDA possessed a uniform spherical‐like morphology, and the outer layer could be attributed to the PDA coating. The modification with PDA not only improved the stability of CaO_2_ and prevented the premature leakage of the L‐Arg, but also enabled good photothermal performance (Figure 15C–E). Specifically, the acidic pH‐induced PDA/CaO_2_ degradation led to the production of NO, about 39.89 µm of NO was generated after incubating LA‐CaO_2_@PDA with pH 6.0 PBS for 24 h. As reflected in the fluorescence intensities, the abundant NO formation in the LA‐CaO_2_@PDA group was due to the large amount of H_2_O_2_ produced by CaO_2_ in the acidic lysosome, while the massive Ca^2+^ retention in the LA‐CaO_2_@PDA + Laser group illustrated that photothermal‐induced oxidative stress could synergize with L‐Arg‐mediated NO to facilitate Ca^2+^ retention in cells (Figure 15F,G). As expected, the LA‐CaO_2_@PDA + Laser group induced the most serious cell damage because of the excellent collaborative effect of intracellular Ca^2+^ overload, NO toxicity, and PTT (Figure 15H). As shown in Figure 15I,J, excessive Ca^2+^ and oxidative stress caused severe mitochondrial dysfunction and membrane lipid peroxidation which were further promoted by photothermal effects. These combined features contributed to ICD, resulting in the surface exposure of CRT, extracellular release of HMGB1, and secretion of ATP (Figure 15K,L). In vivo experiments revealed that the tumor inhibition rate of LA‐CaO_2_@PDA + Laser reached 98.2%, and the powerful immune response could inhibit the lung metastases of 4T1 tumors (Figure 15M–O). Based on the bilateral subcutaneous 4T1‐tumor‐bearing mouse model, it was found that LA‐CaO_2_@PDA + Laser exhibited the strongest inhibitory effects against the growth of both primary and distant tumors (Figure 15P,Q). The robust immune response was characterized by the dramatic increase in DC maturation and an impressive elevation of tumoral infiltrating CD4^+^ helper and CD8^+^ cytotoxic T cells as well as Tregs in primary tumors (Figure 15R–U). This work not only provides an effective combinational treatment method but also inspires a novel paradigm for antitumor IT.

### 4.6 PTT/GT/FT/IT

FT is a form of regulated cell death characterized by the accumulation of Fe‐dependent lipid peroxides. It is distinct from other forms of cell death, such as apoptosis or necrosis, and has emerged as a distinct mechanism with its own set of biochemical and morphological features.^[^ ^222^, ^223^ ^]^ Over the past years, FT has gained attention as a potential therapeutic target, particularly in cancers with high levels of oxidative stress and altered lipid metabolism. The accumulation of lipid peroxides occurs due to compromised antioxidant defenses and impaired activity of enzymes involved in lipid metabolism such as glutathione peroxidase 4 (GPX4) and ferroptosis suppressor protein 1 (FSP1).^[^ ^224^, ^225^, ^226^ ^]^

**FIGURE 16:**
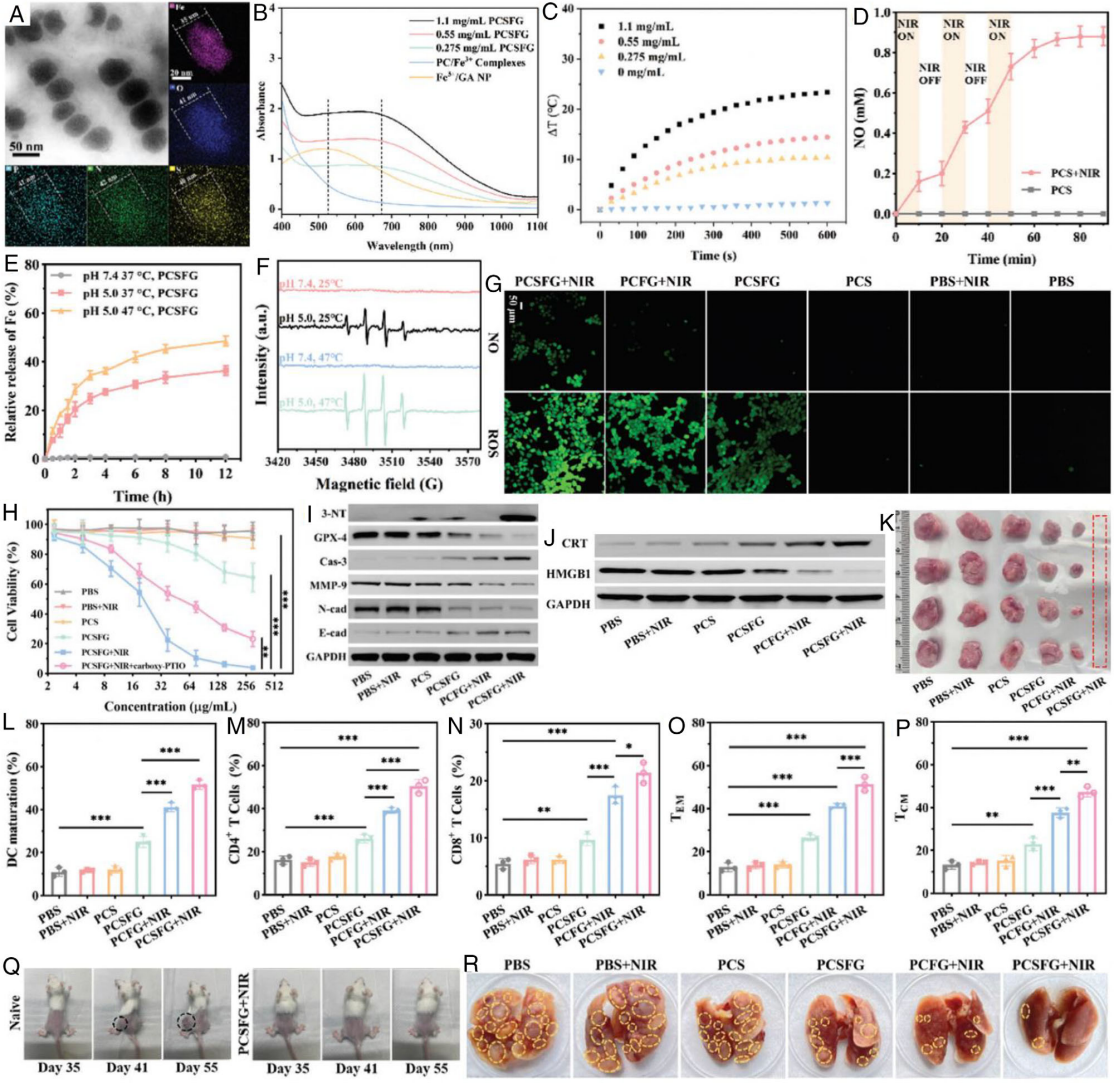
(A) HRTEM image (inset) and 2D elemental mapping (Fe, O, S, N, P) of PCSFG. (B) UV–vis spectra of PC/Fe^3+^ complexes, Fe^3+^/GA NP and PCSFG. (C) Temperature variation curves of PCSFG under the NIR irradiation (808 nm, 1.0 W cm^−2^, 10 min). (D) The switchable NO release from PCSFG. (E) Release profiles of Fe ions from PCSFG under different conditions. (F) ESR spectra of PCSFG when incubated in 20 × 10^−3^ m H_2_O_2_ under different conditions. (G) Fluorescent images of 4T1 cells stained with DAF‐FM DA and DCFH‐DA upon different treatments (*n* = 3, *p ≤ 0.05, ***p ≤ 0.001). (H) Cytotoxicity of PCSFG incubated with 4T1 cells (*n* = 6). (I) Western blot analysis of 3‐NT, GPX‐4, Cas‐3, MMP‐9, E‐cad, N‐cad under different treatments. (J) Western blot analyses of CRT and HMGB1 under different treatments. (K) Representative photographs of tumors after different treatments. (L) Quantification analysis of mature DCs (CD80^+^CD86^+^ gated on CD11c^+^ DCs) in lymph nodes on day 3 after different treatments. Quantification analysis of (M) CD4^+^ and (N) CD8^+^ T cells (gated on CD3^+^ T cells) in tumors on day 3 in different groups (*n* = 3, *p < 0.05, **p < 0.01, ***p < 0.001). Quantification analysis of (O) T_EM_ and (P) T_CM_ cells in spleens. (Q) Representative photographs of naive and rechallenged groups (Mean ± SD, *n* = 3). (R) Representative photographs of lung tissues on day 21 post‐treatment. Reproduced with permission.^[^ ^229^ ^]^ Copyright 2023, Wiley‐VCH GmbH.

Combining PTT/NO gas with FT‐inducing agents can synergistically increase tumor cell eradication, as each modality targets distinct pathways and mechanisms of cell death. The resultant oxidative atmosphere is able to modulate immune‐suppressive factors and promote ICD, leading to the release of tumor antigens and danger signals that stimulate the immune system.^[^ ^227^, ^228^ ^]^ He et al. recently constructed a multifunctional polypeptide coordinate nanocomposite (denoted as PCSFG) with photothermal‐induced NO/Fe^3+^ ions release, which could synergistically kill cancer cells and highly prohibit metastatic 4T1 cell invasion and migration via PTT/GT/FT/IT.^[^ ^229^ ^]^ The PCSFG was rationally designed by coordinating a cytocompatible methacryloyloxyethyl phosphorylcholine and NO co‐grafted poly(d,l‐cysteine) (PCS) with Fe^3+^ ions under the assistance of natural gallic acid. As shown in Figure 16A,B, the obtained PCSFG possessed uniform spherical morphology and Fe‐gallic acid coordination endowed it with decent NIR absorbance for potential PTT. After irradiation by an 808 nm laser (1 W cm^−2^) for 10 min, the PCSFG dispersion (1.1 mg mL^−1^) exhibited a temperature increment of 23.4°C and the PCE was calculated to be 57.8% (Figure 16C). Such photothermal effects could induce NO production by breaking the S─NO bonds and accelerate the release of Fe^3+^ ions under acidic conditions as well as promote the Fenton reaction to generate more •OH (Figure 16D–G). Notably, the PCSFG with laser treatment gave a cell viability below 5%, indicating highly effective cancer cells‐killing activity due to the combined effect of NO‐FT‐PTT (Figure 16H). Western blot was then used to measure the expression levels of 3‐nitrotyrosine (3‐NT), GPX‐4, Caspase‐3, MMP‐9, epithelial cadherin (E‐cadherin), and neural cadherin (N‐cadherin). The results of PCSFG + Laser group verified the presence of NO radicals, FT, and apoptosis in 4T1 cells, tumor invasion and metastasis inhibition, respectively (Figure 16I). Besides, the FT, FT/PTT, and FT/GT/PTT groups caused a progressive increase of CRT but a gradual decline of HMGB1 in contrast to the PBS group, which evidenced that the triple‐modal treatment induced the strongest ICD effects (Figure 16J). Accordingly, tumor growth was remarkably suppressed with a 100% tumor inhibition rate after PCSFG + Laser treatment (Figure 16K). In vivo immune responses were also demonstrated by significant DCs maturation, CD4^+^ and CD8^+^ T cells activation (Figure 16L–N). Excitingly, the PCSFG + Laser group obviously elicited the immune memory effect of T cells, resulting in an increased proportion of central and effector memory T cells (T_CM_ and T_EM_) to prevent tumor recurrence (Figure 16O,P). By rechallenging the PCSFG + Laser‐treated mice with 4T1 cells on day 35 after the first treatment, all of the mice were found to reject the growth of 4T1 tumors until day 55, while the naive mice displayed fast growth (Figure 16Q). In addition, the nodules in the PCSFG + Laser group were dramatically suppressed (≈90%), confirming its good tumor antimetastasis ability (Figure 16R). This work opens up a new avenue for the development of clinically related polypeptide nanoparticles with amplified ICD and antitumor immune memory effects.

### 4.7 PTT/GT/CDT/IT

By cooperating PTT/GT with CDT, multiple mechanisms of cell death are engaged, leading to enhanced tumor cell killing and improved treatment outcomes. Additionally, PTT/NO gas and CDT can modulate the TME by inducing oxidative stress, altering the redox balance, and promoting ICD.^[^ ^230^ ^]^ ICB further unleashes the full potential of the immune response by blocking inhibitory signals, allowing for enhanced tumor cell recognition and destruction by immune cells. Therefore, the combination of PTT/GT with CDT and IT has the potential to provide long‐lasting protection against tumor recurrence and metastasis.^[^ ^231^ ^]^

Cancer‐associated fibroblasts (CAFs) are one of the most abundant stromal cells in TME, playing a crucial role in supporting tumor growth and progression due to their tumor‐promoting and immunosuppressive functions.^[^ ^232^, ^233^ ^]^ Therefore, overcoming the immunosuppressive effects of CAFs and improving immune cell infiltration into the tumor is an active area of research. Shen et al. prepared an injectable hydrogel by chelating mercaptohyaluronic acid with Cu^2+^ ions, which was used to encapsulate antibodies of PD‐L1 and nitrosoglutathione (GSNO) for enhanced IT.^[^ ^234^ ^]^ The injectable hydrogel exhibited a persistent photothermal effect (PCE 49.7%) due to Cu‐mediated NIR laser absorption, leading to the destruction of cancer cells. Besides, the Cu^2+^ from injectable hydrogel could be reduced to Cu^+^ by high expression of GSH under acidic TME, performing a Fenton‐like reaction to produce cytotoxic •OH for CDT. Meanwhile, the increased temperature and high GSH level triggered NO generation from GSNO for GT. These combination therapies could significantly amplify the ICD of tumor cells, which could promote the maturation of DCs and, in turn, activate T cells and other immune cells. More interestingly, the activated CAFs were found to be more sensitive to NO gas than CDT and PTT. The death of CAFs reduced the secretion of TGF‐β, preventing the recruited monocytes at the tumor site to differentiate into M2‐type macrophages. In addition, the ECM was also degraded by the depletion of CAFs, which increased the infiltration of cytotoxic T cells. With the loss of Cu^2+^, the hydrogel collapsed and released anti‐PD‐L1 to increase the IT of recruited cytotoxic T cells. This work illustrates the promising strategy to enhance IT via PTT/GT/CDT‐primed ICD and NO regulation of CAFs‐facilitated ICB.

## 5 CONCLUSIONS AND PERSPECTIVES

During the past decade, PTT has emerged as a promising paradigm in the field of cancer theranostics. To further improve the curative outcome and avoid defects, the integration of NO‐based GT with PTT has gained significant attention in recent years. NO gas‐assisted PTT is a non‐invasive treatment modality that minimizes damage to surrounding healthy tissues and reduces the risk of complications associated with invasive procedures. The localized hyperthermia generated by PTT can quickly destroy cancer cells and lead to immediate tumor shrinkage, while NO can diffuse within the tumor and exert its cytotoxic effects specifically on cancer cells. More importantly, NO gas is able to inhibit the protective autophagy and HSP expression of cancer cells, which are the main obstacles to PTT. By targeting multiple pathways and mechanisms simultaneously, the combination of PTT/GT with other therapeutic modalities can improve treatment efficacy and overcome resistance. Specifically, the photothermal effect and hypoxia relief originated from PTT and NO gas are beneficial to O_2_‐dependent therapies like CHT, PDT, and RT, as well as catalysis‐based approaches like CDT and ST. Additionally, these cooperative treatment modalities are capable of modulating immunosuppressive TME, augmenting the immune response against cancer cells for better antitumor activity.

This work reviews the advances of photothermal/NO‐generating nanomedicines for cancer therapy. It begins by presenting various types of NO donors and precursors, including photothermal‐, ROS‐, light‐, GSH‐, and ultrasound‐senstive ones, in the context of dual‐modal PTT/GT. Among them, the combination of inorganic PTAs (e.g., Au nanorods, CuS nanoplates, Bi_2_S_3_ nanorods, 2D Nb_2_C, and NMOF) and inorganic PTAs (e.g., NA1020 dye, PFTDPP SPNs, and M‐PDA) with thermal‐sensitive NO donors such as RSNOs, BNN, or NONOates is emphasized. Then the incorporation of other treatment modalities such as CHT, PDT, ART, RT, and IT to achieve triple‐modal therapy (e.g., PTT/GT/CHT, PTT/GT/PDT, PTT/GT/ART, PTT/GT/RT, and PTT/GT/IT). Subsequently, tetra‐modal therapies like PTT/GT/CHT/PDT, PTT/GT/CHT/CDT, PTT/GT/PDT/IT, PTT/GT/ST/IT, PTT/GT/Ca^2+^ overload/IT, PTT/GT/FT/IT, and PTT/GT/CDT/IT are included. For better understanding, the above‐mentioned nanoagents have been summarized in terms of materials, PCE, tumor model, biomedical applications, and laser wavelength and density (Table 1). Though inspiring achievements have been obtained via photothermal/NO‐based therapies, several key issues remain unresolved before clinical applications (Table 2).

**TABLE 1:** Summary of different photothermal/NO‐generating nanoplatforms for cancer theranostics.

Material	PCE	Tumor model	Biomedical applications	Laser wavelength and density	Reference
PEG‐PAu@SiO_2_‐SNO	—	MCF‐7 tumor	PTT/GT	808 nm; 1 W cm^−2^	[95]
CuS‐PEI‐TPP	41.80%	4T1 tumor	PTT/GT	1064 nm; 1 W cm^−2^	[96]
BNN‐Bi_2_S_3_	33.7%	BEL‐7402 tumor	PTT/GT	808 nm; 0.35 W cm^−2^	[^97^]
Nb_2_C‐MSNs‐SNO	39.09%	4T1 tumor	PA imaging and PTT/GT	1064 nm; 1 W cm^−2^	[51]
NA1020‐NO@PLX	61%	143B tumor	NIR‐II fluorescence imaging and PTT/GT	1064 nm; 0.5 W cm^−2^	[104]
PFTDPP‐SNAP	48%	MCF‐7 tumor	NIR‐II NIR fluorescence/PA imaging and PTT/GT	808 nm; 1 W cm^−2^	[105]
M/B@R	23.8%	4T1 tumor	PTT/GT	808 nm; 1.5 W cm^−2^	[106]
CPA	72%	4T1 tumor	PTT/GT	808 nm; 1 W cm^−2^	[117]
P‐NO	42.3%	4T1 tumor	NIR fluorescence/PA imaging and PTT/GT	660 nm; 1 W cm^−2^	[108]
PpRE@PEG‐PpIX	—	MCF‐7/ADR tumor	PTT/GT	637 nm; 0.7 W cm^−2^	[109]
CP‐bF@PEG	79.9%	4T1 tumor	NIR‐II fluorescence imaging and PTT/GT	1064 nm; 1 W cm^−2^	[121]
mSZ@PDA‐NO	18.7%	H22 tumor	PTT/GT	—	[111]
MCSN‐SNO/DOX	—	Hep‐G2/ADR tumor	PTT/GT/CHT	1064 nm; 1 W cm^−2^	[140]
IGN Lipo	—	MDA‐MB‐231 tumor	PTT/GT/CHT	808 nm; 1 W cm^−2^	[141]
CSLPC	50.79%	4T1 tumor	NIR fluorescence/NIR‐II PA imaging and PTT/GT/PDT	1060 nm; 1 W cm^−2^	[151]
EArg	41.7%	4T1 tumor	NIR fluorescence imaging and PTT/GT/PDT	660 nm; 150 mW cm^−2^	[155]
IA‐PEP	15.93%	MCF‐7 tumor	PTT/GT/PDT	808 nm; 1 W cm^−2^	[157]
A‐AuNRs@MSN‐SNO	—	HepG2 cells	PTT/GT/ART	808 nm; 2 W cm^−2^	[165]
P(IR/BNN6/AIPH)@Lip‐RGD	—	4T1 tumor	NIR fluorescence imaging and PTT/GT/ART	1064 nm; 0.8 W cm^−2^	[89]
Bi‐SNO	15.5%	U14 tumor	CT imaging and PTT/GT/RT	808 nm; 0.8 W cm^−2^	[174]
Cu_2_O/BNN6@MSN‐Dex	23.56%	HT29 tumor	PA imaging and PTT/GT/IT	808 nm; 1.5 W cm^−2^	[188]
SPNaiB	—	4T1 tumor	PTT/GT/IT	1064 nm; 1 W cm^−2^	[193]
NPS_D‐IR_	—	MCF‐7/R tumor	PTT/GT/PDT/CHT	808 nm; 1.3 W cm^−2^	[197]
PIDA	13.8%	K562/ADR tumor	PTT/GT/PDT/CHT	808 nm; 0.5 W cm^−2^	[198]
MAPRF	—	HeLa cells	MR imaging and PTT/GT/CHT/CDT	808 nm; 1.5 W cm^−2^	[205]
CMH‐OBN	—	4T1 tumor	MR imaging and PTT/GT/PDT/IT	808 nm; 1 W cm^−2^	[148]
C‐NO@PEG	29.7%	4T1 tumor	PTT/GT/PDT/IT	1064 nm; 0.8 W cm^−2^	[209]
M@BPAG	—	GL261‐Luc tumor	PTT/GT/ST/IT	808 nm; 2 W cm^−2^	[216]
LA‐CaO_2_@PDA	—	4T1 tumor	PTT/GT/Ca^2+^ overload/IT	808 nm; 1 W cm^−2^	[221]
PCSFG	57.8%	4T1 tumor	PTT/GT/FT/IT	808 nm; 1 W cm^−2^	[229]
Injectable hydrogel	49.7%	4T1 tumor	PTT/GT/CDT/IT	808 nm; 0.8 W cm^−2^	[234]

**TABLE 2:** A comparison of different NO donors/precursors and related NO‐releasing mechanisms.

NO donors/precursors	Triggers	NO‐releasing mechanisms	Reference
─SNO	Photothermal	Remarkable hyperthermia from photothermal conversion caused the breakage of S─NO bonds	[51, 140, 165, 229]
SNAP	Photothermal	Low‐temperature PTT process broke the S─NO bonds	[104, 105]
BNN6	Photothermal	Photothermal‐induced N─NO bonds breakage	[89, 106, 188]
N‐nitrosamines	Light	Green light‐induced conversion of electron‐withdrawing group N‐nitrosamines into an electron‐donating NH group with NO simultaneously generated	[108]
RRS	Light	PpIX ring directly absorbed light energy and transfered it to the nitrosyl group to release NO	[109]
NTFA	Ultrasound‐chargeable PL	Under PL irradiation, an optical rearrangement of the nitro‐nitrite occurred and generated phenoxy and NO radicals	[111]
L‐Arg	H_2_O_2_	H_2_O_2_ promoted oxidation of the guanidine nitrogen in L‐Arg to generate NO and L‐citrulline	[117, 193, 216, 221]
Benzofuroxan	GSH	The nucleophilic thiol group of GSH was added to the conjugated structure of furoxan to substitute NO	[121, 148]
NONOate	Low pH	NONOate was not stable and underwent a proton‐catalyzed dissociation pathway to produce NO under physiological conditions	[141]
L‐Arg	^1^O_2_	The abundant ^1^O_2_ generated from the photodynamic process was able to oxidize the L‐Arg to produce NO	[151, 155, 156, 198]
─SNO	X‐ray	X‐ray radiation broke down the S─NO bonds	[174]
─ONO_2_	GSH/photothermal	Thiol‐mediated NO release from nitrate esters was due to an initial two‐electron reduction followed by a thiolate nucleophilic attack on the nitrate group, via a sulfenate intermediate; the photothermal effect was also able to boost the NO release	[197, 209]
Ru‐NO	Light	Under light irradiation, a photoelectron was promoted from the π orbital of the metal ion to the π anti‐bond orbital of NO, resulting in the rapid release of NO	[205]
GSNO	GSH/photothermal	GSH underwent transnitrosation with SNO to substitute NO from SNO, and photothermal‐induced S─NO bonds breakage to boost NO generation	[234]

PTT is dependent on the absorption of light by PTAs to generate heat and induce therapeutic effects, but the penetration depth of light in tissues is limited, particularly in deep‐seated tumors. Thus, the development of NIR‐II PTAs with high PCE is in urgent need, which ensures more safe powder density and longer wavelength of laser as well as a lower administration dosage. Achieving targeted delivery of photothermal/NO nanosystems to tumor sites is crucial to minimize off‐target effects and maximize therapeutic efficacy. Understanding the biodistribution kinetics of the nanosystems and cutting down their accumulation in non‐target organs or tissues is crucial for safe and effective therapy. For example, necessary surface functionalization with PEG or tumor‐specific molecules not only can promote biocompatibility, but also blood circulation time as well as tumor accumulation. Achieving the optimal therapeutic dosage of NO gas may be challenging. Too low a concentration may not provide sufficient therapeutic effect, while excessive concentrations could lead to unwanted side effects, such as systemic hypotension or cytotoxicity. Careful monitoring and control of NO dosage are necessary to ensure a safe and effective treatment. Tumors are known for their heterogeneity in terms of cellular composition, genetic mutations, and microenvironment. This heterogeneity can lead to variations in the response to PTT/GT among different tumor regions or even within the same tumor. Some areas may be more resistant to the therapy, limiting its overall effectiveness. Triple‐modal and tetra‐modal approaches of photothermal/NO nanosystems indeed offer synergistic benefits, but they may also present challenges in terms of treatment optimization and potential adverse interactions between different modalities. Finding the right balance and optimizing the parameters for each modality is important to maximize therapeutic efficacy with minimized side effects. Nanomaterials used in such nanosystems must be biocompatible by tuning their sizes, surface charges, compositions, and stabilities to decrease adverse reactions and toxicity. The presence of nanomaterials in the body can interact with the immune system and modulate immune responses. This can be advantageous for enhancing antitumor immune responses, but it can also lead to unintended immunomodulation or immune dysregulation. Understanding the interactions between the nanosystems and the immune system is of significance to optimize therapeutic outcomes and weaken immune‐related adverse effects. Biodegradability and efficient clearance mechanisms are desirable to reduce potential long‐term accumulation and associated toxicity after the completion of therapy. Hence, long‐term studies are necessary to assess the potential cumulative effects and ensure the safety of these nanosystems over extended treatment periods. Meanwhile, simple and cost‐effective synthetic methods for large‐scale preparation of such novel nanoplatforms under mild conditions should be explored. Although preclinical studies have shown promising results, the clinical evidence for photothermal/NO‐based therapies is still limited. Large‐scale clinical trials are needed to evaluate the safety, efficacy, and long‐term outcomes of this combination therapy in human patients.

## CONFLICT OF INTEREST STATEMENT

The authors declare no conflicts of interest.
